# Structural and solvent modulation of symmetry-breaking charge-transfer pathways in molecular triads†

**DOI:** 10.1039/d4sc05419a

**Published:** 2024-09-26

**Authors:** Chinju Govind, Evangelos Balanikas, Gana Sanil, Daniel T. Gryko, Eric Vauthey

**Affiliations:** a Department of Physical Chemistry, University of Geneva 30 Quai Ernest-Ansermet CH-1211 Geneva 4 Switzerland eric.vauthey@unige.ch; b Institute of Organic Chemistry, Polish Academy of Sciences 01-224 Warsaw Poland

## Abstract

Whereas the photoinduced charge-transfer properties of electron donor–acceptor dyads are now well understood, those of symmetric conjugated architectures containing several identical donor–acceptor branches have started to be scrutinised much more recently. Here, we report on our investigation of the charge-transfer dynamics of a series of formally centrosymmetric triads consisting of a quadrupolar dihydropyrrolopyrrole core substituted with two identical diphenylethynyl lateral branches. Using a combination of time-resolved electronic and vibrational spectroscopies, we show that these molecules exhibit rich excited-state dynamics, which includes three different types of symmetry-breaking charge-transfer processes depending on the nature of the end substituents on the core and branches as well as on the solvent: (i) excited-state symmetry breaking within the core; (ii) charge transfer from the core to one of the two branches; (iii) charge transfer between the two branches. This investigation illustrates how the excited-state properties of symmetric conjugated molecules, including the nature and location of the exciton, can be controlled by fine tuning structural as well as environmental parameters.

## Abstract 2

Ultrafast vibrational spectroscopy reveals that centrosymmetric dihydropyrrylopyrrole-based triads can follow three distinct photo-induced symmetry-breaking charge-transfer pathways, depending on the substituents and solvent.

## Graphical abstract

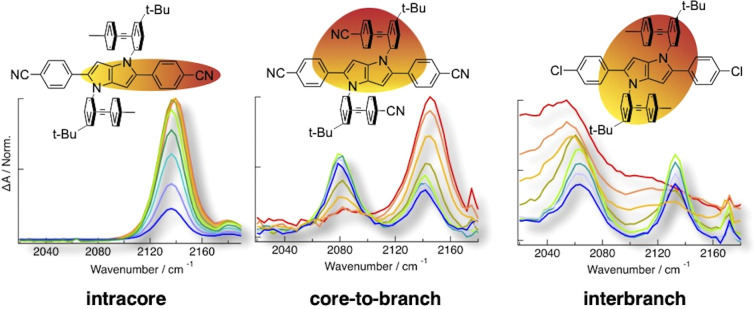

## 1 Introduction

Thanks to the immense bulk of experimental work performed over the past decades on electron donor–acceptor (D–A) dyads,^1–18^ we have reached a deep understanding of intramolecular photoinduced electron transfer (ET) processes. In these dyads, the D and A subunits, hence the direction of the charge flow, can be clearly identified. Over the past few years, there has been a growing interest for symmetric molecular architectures with two or more energetically equivalent photoinduced charge-transfer (CT) pathways.^19^ CT along one of these paths results in a breaking of the symmetry of the electronic structure. Several types of symmetry-breaking (SB) CT processes were identified so far. One of them consists in a charge separation (CS) between two identical molecules after the photoexcitation of one. According to polarised transient electronic absorption measurements, the origin of the SB is not the photoexcitation itself but rather the instantaneous asymmetry of the solvent field around the two molecules.^20^ Such SB-CS processes are being reported for a growing number of molecules, including perylene,^20–22^ perylene mono- and diimides,^23–27^ pyrene,^28^ anthracene and dipyrrin derivatives,^29–31^ as well as naphthalenediimides,^32,33^ in polar environments. The other type of systems undergoing SB-CT consists of a chromophoric D subunit linked to two or more identical A moieties, or *vice versa*. When the electronic coupling between the constituents is weak, excitation is localised on the central chromophoric unit and SB-CS takes place with one of the surrounding A or D moieties.^16,34–38^ By contrast, when the D and A groups are connected through a conjugated bridge, the coupling between the constituents is sufficiently large for the electronic excitation to be delocalised.^39–53^ In non-polar environments, the excited state is generally multipolar and symmetric as it is distributed evenly over the whole molecule. In polar solvents, a so-called excited-state (ES) SB takes place, resulting in a localisation, at least partial, of the excitation on a single D–A branch. As a consequence, the excited state acquires a dipolar character. Such ES-SB occurs when the gain in solvation energy upon localisation exceeds the loss of interbranch excitonic coupling.^54–56^

**Fig. 1:**
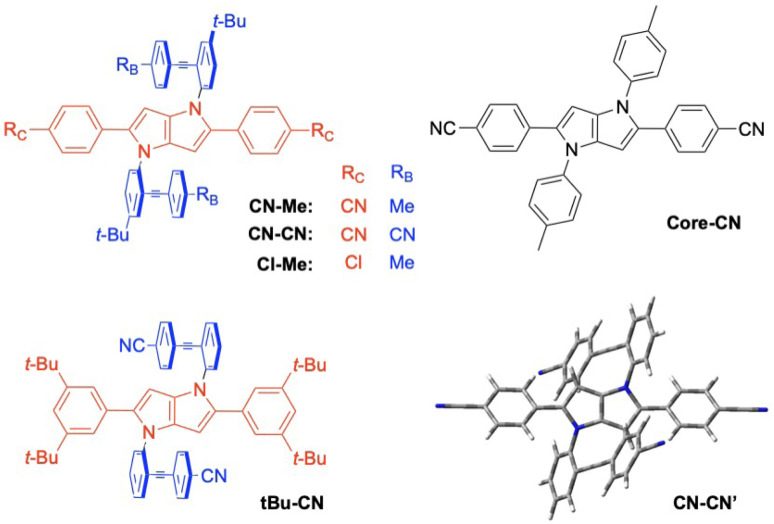
Structure of the triads and of the DHPP analogue Core-CN. The optimised geometry of an analogue of CN–CN without the *tert*-butyl groups is shown on the bottom right.

Here, we report on our investigation of the photoinduced CT dynamics in a series of formally centrosymmetric triads consisting of a quadrupolar dihydropyrrolopyrrole (DHPP) chromophoric core with two diphenylethynyl (DPE) branches attached on the nitrogen atoms (Fig. 1).^57^ The electron donating and withdrawing strength of the subunits is tuned by varying the terminal substituents on both the core and the branches. The presence of two different types of IR-active probes, *i.e.*, cyano groups and carbon–carbon triple bonds, at different positions offers a unique opportunity to monitor the location of the excitation in the molecule and to detect SB-CT. Using a combination of time-resolved electronic and vibrational spectroscopies, we show that various SB-CT processes occur upon photoexcitation of these dyes, depending on the substituents and the solvents. This investigation illustrates how the charge distribution in symmetric conjugated molecular architectures can be controlled by fine-tuning structural and environmental properties.

## 2 Results

### 2.1 Stationary spectroscopy, ns fluorescence and quantum-chemistry calculations

**Fig. 2:**
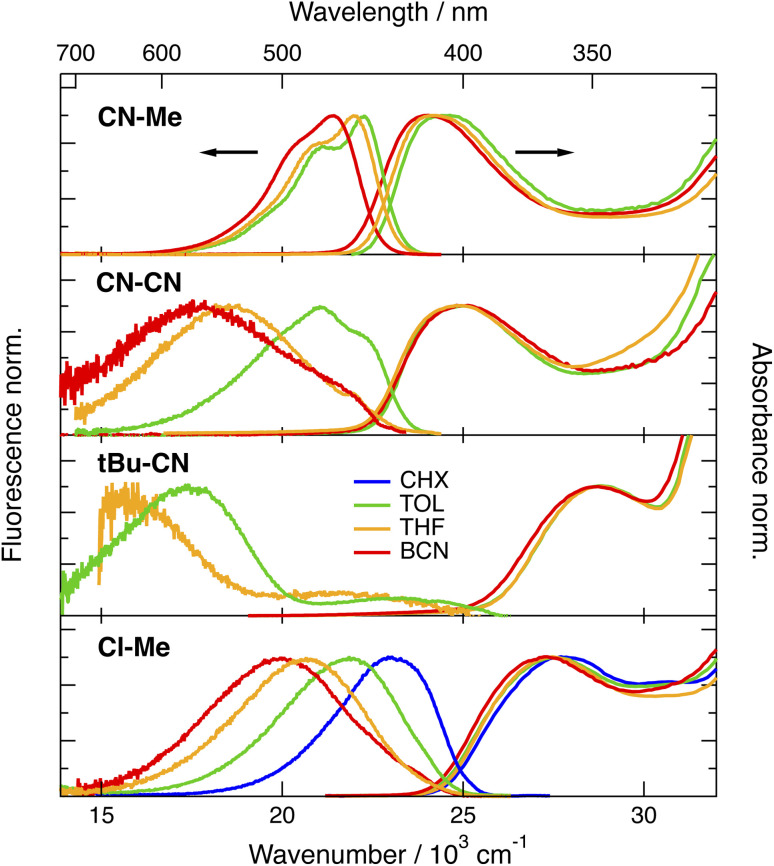
Stationary electronic absorption and fluorescence spectra (400 or 355 nm excitation) recorded with the triads in various solvents (CHX: cyclohexane; TOL: toluene; THF: tetrahydrofuran; BCN: benzonitrile).

The stationary absorption spectra of all four dyes are dominated by a broad and structureless band peaking around 400 nm for CN–Me and CN–CN and between 350 and 360 nm for the two others, as well as by a second more intense band at higher energy (Fig. 2). By comparison with the absorption spectrum of the analogue molecule without the DPE branches (Core-CN, Fig. 1) reported in ref. 47 and 58, the lowest energy band can be assigned to the DHPP core and the other to the DPE branches. Very little solvatochromism is observed, as expected for centro-symmetric molecules. The fluorescence spectrum, quantum yield, solvatochromism and lifetime of CN–Me are very similar to those of the Core-CN analogue (Table S3†). By contrast, the other dyes are much less fluorescent with quantum yields amounting to a few percent or less,^57^ and exhibit markedly stronger solvatochromism. Dual emission is observed with *t*Bu-CN with the lowest energy band shifting above 700 nm in highly polar solvents. Despite the small fluorescence quantum yields, the fluorescence lifetimes of these three dyes measured around the band maximum are relatively long, between 3 and 8 ns (Fig. S4†). These values correspond to small radiative rate constants, *k*_rad_, of the order of 10^6^–10^7^ s^−1^, relative to 7.6 × 10^8^ s^−1^ for CN–Me (Table S3†).

To try rationalising these stationary spectra, quantum-chemical calculations were carried out at the density functional theory (DFT) and time-dependent (TD) DFT levels (CAM-B3LYP/6-31g(d,p)). They were performed in the gas phase, with the geometry restricted to *C*_i_ symmetry, and with the *tert*-butyl groups of CN–Me, CN–CN and Cl–Me replaced by hydrogen atoms. In agreement with previous calculations with CN–Me and CN–CN,^57^ the DPE branches are oriented by about 64° out of the plane defined by the DHPP core for all four compounds (Fig. 1). The structural flexibility of these molecules at room temperature was tested with CN–CN for the core-branch angle, as well as for the torsional angle between the two phenyl groups of a DPE branch and between a phenyl group and the pyrrolopyrrole centre of the core. The results, presented in Fig. S1† suggest that, at room temperature, both the core-branch angle and the core torsional angle can fluctuate within approximately ±15°. On the other hand, the DPE branches exhibit significantly higher flexibility, ±25°, as well known for other phenylethynyl based molecules.^59–61^ As a consequence, these dyes are not strictly centrosymmetric at room temperature.

In agreement with ref. 57 and with the stationary spectra, the TD-DFT calculations predict the S_1_ ← S_0_ transition of CN–Me to be localised on the core. The S_2_ ← S_0_ transition is 0.45 eV higher and is characterised by a strong CT character with no oscillator strength, *i.e.*, *f* = 0. It is associated with a one-electron transition from the HOMO, localised on the core, to the LUMO centred on the branches (Fig. S2†). For CN–CN and Cl–Me, the S_1_ ← S_0_ transition has some additional CT character towards the branches, whereas the S_2_ ← S_0_ transition is also of a pure core-to-branch CT character. However, it lies closer to the S_1_ state, namely, 0.12 and 0.20 eV, respectively (Fig. S2†). Finally, for *t*Bu-CN, the calculations predict the S_1_ and S_2_ states to be nearly degenerate and of pure CT character, and the next state, S_3_, to lie 0.6 eV above and to have contributions from several one-electron transitions, including one involving mostly the branches (Fig. S2†). Considering the *C*_i_ symmetry imposed in these calculations, the CT states should be purely quadrupolar. Such quadrupolar CT states can be expected to be unstable in polar environments, especially in view of the structural flexibility of these molecules.

Based on these calculations, the strong changes in the fluorescence properties observed upon going from CN–Me to the three other molecules can be explained by a change in the nature of the S_1_ state, namely a locally-excited (LE) state centred on the core for CN–Me and a CT state for the others. Whereas calculations predict dark CT transitions, CT emission is visible, although with a small *k*_rad_, most probably because of the flexibility of the molecules and, thus, the departure from a strictly centrosymmetric structure.

### 2.2 Time-resolved spectroscopy

#### 2.2.1 Transient electronic absorption

**Fig. 3:**
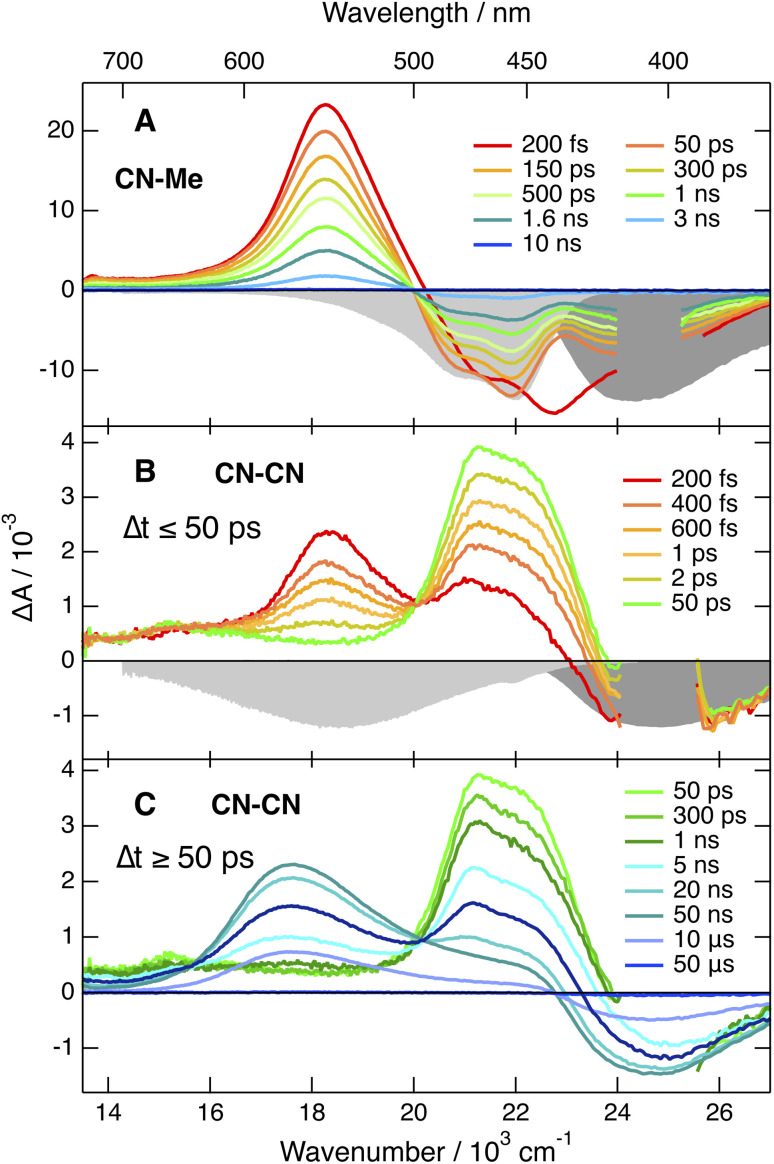
Electronic transient absorption spectra recorded at various time delays after 400 nm (<2 ns) or 355 nm excitation (>2 ns) of (A) CN–Me and (B and C) CN–CN in tetrahydrofuran, together with the negative stationary absorption and stimulated emission spectra (grey shading).

Transient electronic absorption (TA) measurements were carried with all triads in weakly polar toluene (TOL), medium polar tetrahydrofuran (THF) and highly polar benzonitrile (BCN). The TA spectra recorded upon excitation of CN–Me resemble those reported previously with Core-CN,^47^ namely, they are dominated by a positive band centred around 550 nm attributed to S_*n*>1_ ← S_1_ excited state absorption (ESA) and a negative band below 500 nm, assigned to both stimulated emission (SE) and ground-state bleach (GSB) (Fig. 3A, and S5†).

Apart from some early spectral dynamics in the SE region, arising most probably from vibrational/solvent relaxation, these features decay with a ∼1.4 ns time constant to a weak residual spectrum that is hardly visible in THF and not detectable in BCN (Fig. S6†). This residual, which is the most distinct in TOL, where it decays with a 1.6 μs time constant, is attributed to the lowest triplet excited state, T_1_.

The TA spectra recorded with the three other dyes are qualitatively similar to each other but differ significantly from those measured with CN–Me. The spectra obtained with CN–CN are presented in Fig. 3B, C and S7†, while the other are shown in Fig. S9 and S11.† All TA data were analysed globally assuming a series of successive exponential steps. The resulting evolution-associated difference absorption spectra and time constants are presented in Fig. S8, S10 and S12.† The early TA spectra of CN–CN resemble those of CN–Me with the ESA band around 550 nm due to the LE state. However, this band decays in about 4 ps in TOL and <1 ps in THF and BCN, while two bands, a weak one above 600 nm and a more intense below 500 nm, grow concurrently. The band below 500 nm presents a double maximum at 475 and 454 nm in TOL. The feature at 454 nm becomes a shoulder in THF and is no longer visible in BCN. Based on the quantum-chemical calculations, these bands are attributed to a CT state with the electron on a DPE branch and the hole on the DHPP core. Their positions are consistent with the absorption spectra of both the radical anion of DPE,^62^ and the radical cation of a similar DHPP derivative,^63^ which show an intense band in the 450–500 nm region and a weaker one above 600 nm. Both bands decay simultaneously in 5–10 ns in parallel to the rise of a new band at 570 nm, which in turns decays on the 10 μs timescale together with the GSB. This band resembles that measured at late time with CN–Me and is attributed to the T_1_ state, most probably localised on the DHPP core. As the amplitude of the GSB remains mostly unchanged during the decay of the CT bands, a triplet yield close to unity can be deduced.

The TA spectra measured with Cl–Me exhibit similar behaviour (Fig. S11†). However, the LE band is less intense due to its faster conversion to the CT band relative to CN–CN. The shape of the CT band below 500 nm presents also some solvent dependence and decays in a few ns concurrently to the rise of the triplet state band. Even faster LE → CT conversion is observed with *t*Bu-CN, and the LE band is hardly visible with the ∼100 fs time resolution of the experiment (Fig. S9†). The shape of the CT band below 500 nm is also solvent dependent with two maxima in THF and BCN. Contrary to CN–CN and Cl–Me, no triplet ESA band is visible in BCN. This band is present in TOL and THF, but its amplitude is smaller than for CN–CN and Cl–Me, especially in THF.

#### 2.2.2 Time-resolved IR absorption

The above electronic TA data point unambiguously to the occurrence of CT with CN–CN, Cl–Me and *t*Bu-CN. However, they give little information on the nature of this state, although the quantum-chemical calculations suggest a core-to-branch CT state (Fig. S2†). To obtain better insight, we performed time-resolved IR absorption (TRIR) measurements in the –C≡C– and –C≡N stretching region.

**Fig. 4:**
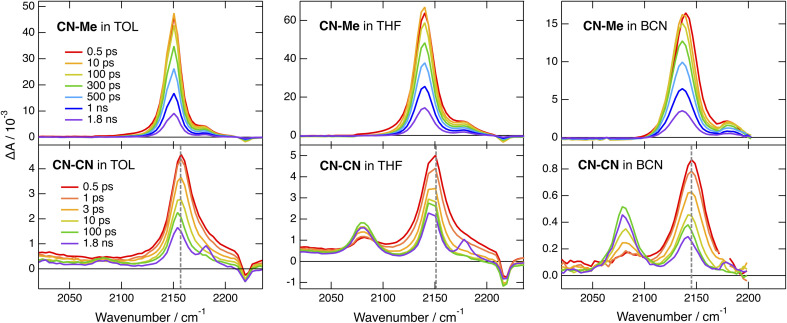
Time-resolved IR absorption spectra recorded at various delays after 400 nm excitation of CN–Me (top) and CN–CN (bottom) in toluene, tetrahydrofuran and benzonitrile. The colour code for the time delays is the same for a given triad. The dashed vertical line in the bottom panels highlights the temporal shift of band.

The TRIR spectra recorded with CN–Me in TOL, THF and BCN exhibit two positive bands, a relatively intense one and a weaker one at higher frequency, additionally to a small negative band at even higher energy (Fig. 4, top). The frequency splitting of the two ESA bands depends on the solvent polarity and increases continuously from 30 to 45 cm^−1^ by going from TOL to BCN. Moreover, the relative intensity of the weaker ESA band increases slightly during the first few ps after excitation. These transient spectra are very similar to those reported with the Core-CN analogue.^47,64^ Therefore, the intense and weak ESA bands are assigned to the antisymmetric and symmetric –C≡N stretching modes of the dye in the LE state, and the negative band is due to the ground-state bleach of the antisymmetric –C≡N stretching mode. The presence of the symmetric –C≡N stretching band reveals that the LE state is not fully centrosymmetric and quadrupolar. Such quadrupolar excited state with a single ESA band was observed with Core-CN in non-polar solvents.^47,64^ ES-SB was found to already take place in weakly polar solvents like TOL. Therefore, the excitation is no longer distributed evenly on the two sides of the molecule and the symmetric –C≡N mode acquires some IR activity. Previous studies on Core-CN revealed that inertial solvent motion is sufficient to break symmetry in less than about 100 fs, *i.e.*, within the instrument response function of the TRIR set-up.^47^ Further SB occurring *via* diffusive solvent motion is responsible for the temporal increase of the relative intensity of the weaker ESA band. Consequently, these TRIR data confirm that photoexcitation of CN–Me results in the population of an excited state localised on the DHPP core. The frequency downshift of the –C≡N stretch in the LE state relative to the ground state is due to weakening of the triplet-bond character upon increasing electronic density.^65,66^ The absence of –C≡C– stretching band in the transient spectra indicates that the electronic density on the DPE branches is not affected by the excitation and point to a weak electronic coupling through the pyrrolic nitrogen atoms. Moreover, these data reveal a lopsided distribution of the excitation on the two terminal cyano groups of the DHPP core, as observed with Core-CN. Furthermore, the increasing frequency splitting of the two ESA bands points to an increasing degree of asymmetry with the polarity of the environment.^47,64,67^

The early TRIR spectra recorded with CN–CN exhibit an intense band around 2160 cm^−1^, similar to that of the –C≡N stretching band of the DHPP core, as well as a weaker band around 2080 cm^−1^ (Fig. 4, bottom). The more intense band decreases partially and undergoes a small frequency downshift during the first few ps, while the 2080 cm^−1^ band grows concurrently. The relative amplitude of these two bands depends on the solvent polarity: the 2080 cm^−1^ band remains much smaller than the other in TOL, reaches about the same intensity in THF and becomes markedly larger in BCN. Both bands decay on the ns timescale in parallel to the rise of a weaker band around 2180 cm^−1^, which, by analogy with a previous study with Core-CN,^64^ can be attributed to the –C≡N stretching mode of the core-localised T_1_ state. The 2080 cm^−1^ band is attributed to the –C≡C– stretching vibration of the CT state. It is at similar frequency as the –C≡C– bands observed previously upon photoexcitation of alkynylated push–pull molecules.^38,68,69^ The band around 2160 cm^−1^ can be assigned to a –C≡N stretching vibration. However, it is not clear whether it is related to the cyano substituents on the DHPP core or to those on the DPE branches.

**Fig. 5:**
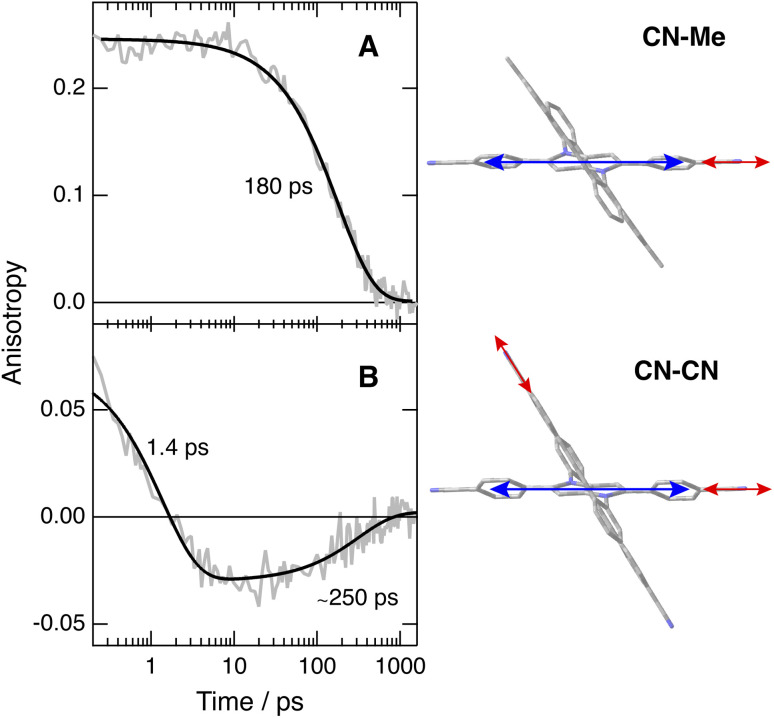
Time dependence of the polarisation anisotropy of the –C≡N ESA band measured after 400 nm excitation of (A) CN–Me and (B) CN–CN in THF and best fits of a mono- and a bi-exponential function. The right panels illustrate the directions of the electronic (blue) and vibrational (red) transition dipoles.

To disentangle the vibrations originating from these cyano substituents, we performed polarised TRIR experiments and measured the polarisation anisotropy of the –C≡N band as a function of time. As control, these measurements were also performed with Core-CN and CN–Me. For these two molecules, the initial anisotropy of the antisymmetric –C≡N band measured in different solvents is between 0.20 and 0.25 (Fig. 5A, S20 and S21†). It decays to zero in a few hundreds of ps, with a time constant increasing with the solvent viscosity, as expected for reorientational motion.^70,71^ These initial anisotropies are smaller than the value of 0.4 expected for a vibrational transition dipole exactly parallel to the S_1_ ← S_0_ electronic transition dipole, but are consistent with that expected for cyano groups oriented along the main molecular axis of the DHPP core.

As illustrated in Fig. S22† and 5B, the anisotropy of the –C≡N band measured with CN–CN in TOL and THF is first positive but evolves within a few ps to a negative value, before decaying to zero in a few hundreds of ps. A positive anisotropy is consistent with a vibrational mode involving the DHPP cyano groups. On the other hand, a negative anisotropy points to an angle of more than 55° between the vibrational and electronic transition dipole. This is what is expected for the vibrational mode involving the cyano groups on the DPE branches. Consequently, the change of sign of the anisotropy directly reflects the LE → CT transition. The frequency downshift of the –C≡N band observed within the first few ps after excitation (Fig. 4, bottom) can be explained by the change in the origin of this band.

To understand why the –C≡N vibration of the core is apparently not visible in the TRIR spectrum of the CT state, we carried out quantum-chemical calculations of the vibrational modes of CN–CN and of its radical cation and anion (Table S1†). The hole of the cation and unpaired electron of the anion are predicted to be located on the DHPP core and on a DPE branch, respectively (Fig. S3†). The core and branch antisymmetric –C≡N stretches of the neutral CN–CN should be only 3 cm^−1^ apart and have similar IR intensities, around 100 km mol^−1^ (Table S1†). On the other hand, the IR intensity of the antisymmetric core –C≡N stretch of the cation should be negligibly small, while that of the branch –C≡N stretch of the anion should amount to 1300 km mol^−1^, similar to the oscillator strength of the –C≡C– stretch.

The TRIR spectra measured with *t*Bu-CN exhibit a behaviour that is very similar to that of CN–CN (Fig. S15 and S16†). The main differences are, first, a faster rise of the –C≡C– band, in agreement with a faster CT and, second, a larger relative amplitude of this band in TOL. Additionally no clear evidence of the triplet state is visible at longer time delays, in agreement with the lower triplet yield deduced from the electronic TA measurements.

**Fig. 6:**
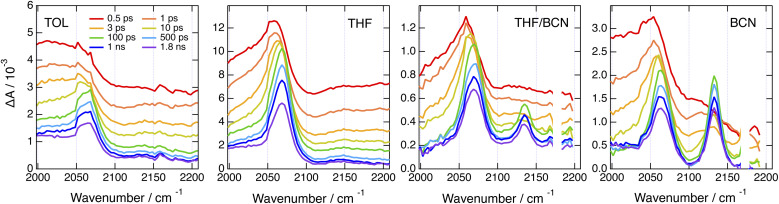
Time-resolved IR absorption spectra recorded at various delays after 360 nm excitation of Cl–Me in solvents of increasing polarity and in a 55 : 65 (v/v) tetrahydrofuran/benzonitrile mixture.

By contrast, significantly different TRIR dynamics are observed with Cl–Me. The early transient spectra exhibit a quasi uniform background, except in BCN where a band around 2070 cm^−1^ can be distinguished (Fig. 6 and S17†). In TOL and THF, this broad spectrum decays almost entirely within a ps and transforms into a spectrum with a single band around 2070 cm^−1^. This band narrows and undergoes a ∼5 cm^−1^ blue shift in a few ps before decaying completely in a few ns. In BCN, the early broad spectrum transforms within 20 ps into a spectrum with two bands of similar intensity at 2070 and 2140 cm^−1^, which then decay concurrently in a few ns.

The single band observed after a few ps in TOL and THF is consistent with the –C≡C– stretching mode of the CT state. As the DHPP core of Cl–Me does not possess any vibrational marker, no TRIR band should be visible in this spectral region when the molecule is in the LE state. Broad structureless TRIR spectra, as those visible here at early time, have also been observed with several multibranched D–A dyes.^72–74^ They were assigned to a mid-IR electronic transition. Each D–A branch of these dyes can be viewed as a chromophore with a CT optical transition. For a A–D–A dye like Core-CN, excitonic coupling between the two D–A sides leads to two delocalised states split by twice the interbranch excitonic coupling, equivalent to the Davidov splitting. Depending on the magnitude of this coupling, optical transition between these two excitonic states can be found in the mid-IR region. This band is characteristic of the delocalised excited state and vanishes completely upon ES-SB.^72–75^ In the case of Core-CN, this transition was found to be above 3400 cm^−1^.^72^ The presence of such electronic transition in the 2000–2200 cm^−1^ region for Cl–Me suggests a smaller excitonic coupling than for Core-CN. This can be explained by the smaller electron withdrawing properties of the two chlorophenyl side-groups of the DHPP core compared to the cyanophenyls of Core-CN. This should lead to a smaller CT transition dipole in each branch of the DHPP core, hence to a smaller excitonic coupling. Therefore, the initial decay of the broad TRIR absorption can be assigned to the occurrence of ES-SB in the LE state, similar to that observed with CN–Me. For CN–CN, this electronic transition should be at higher energy like for Core-CN, and is not visible in this spectral region. The ensuing rise of the –C≡C– band reflects the population of the CT state, with a shift of the electronic density from the core to a branch. The triplet state resulting from the decay of the CT state observed in the electronic TA data is not visible here, although its population is confirmed by the electronic TA measurements (Fig. S11†). This confirms that the T_1_ state is localised on the DHPP core, which, in the case of Cl–Me, does not contain any vibrational marker.

The presence of two bands of similar intensity observed after about 20 ps in BCN is more surprising. The 2070 cm^−1^ band is also visible in TOL and THF and can be similarly assigned to the –C≡C– stretching mode of a DPE branch with an excess electron density. As the only vibrational markers of Cl–Me in this spectral region are associated with the two DPE branches, the second band at 2140 cm^−1^ should also be related to a –C≡C– stretching mode. Therefore, the TRIR spectrum with these two bands is due to an excited state delocalised over the two DPE branches. The 70 cm^−1^ splitting of these bands point to markedly different electronic density on the two DPE branches. Consequently, we attribute this transient spectrum to a state involving a CT from one DPE branch to the other, similar to a SB-CS state between two identical molecules discussed in the introduction. This assignment agrees with quantum-chemical calculations of the –C≡C– stretching frequencies of Cl–Me in the ground state and of its radical ions. The antisymmetric –C≡C– stretches of the anion and cation are predicted to be frequency downshifted by 140 and 8 cm^−1^ relatively to the ground state and to have a similarly larger IR intensities, *i.e.*, around 300 *vs.* 40 km mol^−1^ (Table S2†).

This interbranch CT state is distinct from the core-to-branch CT state found in TOL and THF as well as with CN–CN and *t*Bu-CN, which have stronger electron-withdrawing branches (*vide infra*). The observation of this interbranch state in BCN only could indicate that its population is favoured by a larger solvation energy. To test this, we performed TRIR measurements with Cl–Me in binary THF/BCN mixtures to vary the dielectric constant of the solvent. As illustrated in Fig. 6, S18 and S19,† whereas the 2140 cm^−1^ band is hardly visible in pure THF, its intensity increases continuously with the BCN content of the mixture. Moreover, the its rise time becomes shorter upon increasing the BCN content. This confirms that, in highly polar solvents, the interbranch CT state is stabilised relatively to the core-to-branch CT state.

## 3 Discussion

### 3.1 The nature of the CT states

The above results reveal how the rich and diverse photoinduced CT dynamics of these formally centrosymmetric dyes can be tuned by varying both terminal substituents of the core and branches as well as the environment.

**Fig. 7:**
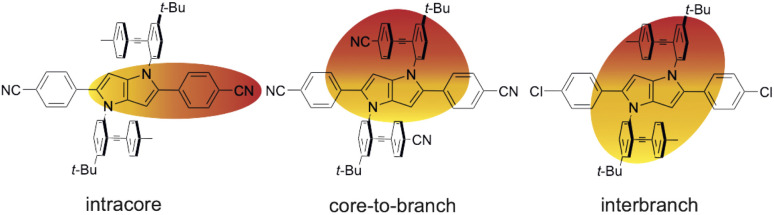
Schematic representation of the three different symmetry-broken charge-transfer states. Increase and decrease of electronic density are represented in red and yellow, respectively.

Essentially, three different types of CT processes leading to a symmetry breaking of the electronic structure are observed (Fig. 7):

(1) Intracore CT. The electronic TA data indicate that optical excitation of these dyes leads to the population of a LE state centred on the DHPP core. According to previous investigations with Core-CN and structurally related DHPP-based linear A–D–A dyes,^47,56,76^ the Franck–Condon excited state is mostly quadrupolar with an even distribution of the excitation on the two sides of the DHPP core. However, ES-SB occurs rapidly already in weakly polar solvents, leading to an asymmetry of the excitation, which increases with the solvent polarity. This ES-SB within the DHPP core is visible here from the presence of the symmetric –C≡N stretching mode (Fig. 4, top) and the decay of the broad mid-IR electronic band (Fig. 6) in the TRIR spectra of the LE state of CN–Me and Cl–Me, respectively. For the other two dyes, CN–CN and *t*Bu-CN, the LE → CT transition is so fast that the early transient spectra already contain contribution from the CT state, inhibiting the detection of ES-SB in the LE state.

(2) Core-to-branch CT. This process is observed with all dyes except CN–Me. Although, we have no direct evidence, this CT should also lead to a breaking of the symmetry. It is indeed doubtful that electronic density is transferred equally from the core to the two DPE branches to give rise to a quadrupolar CT state. As shown previously, solvation energy favours localisation of the charges and a dipolar state over a delocalised quadrupolar state.^55^ Another question that cannot be answered from the data is whether the initial SB of the LE state determines the direction of the core-to-branch CT. Considering that the DPE branches are attached on the two opposite sides of the core, one could envisage that, if the LE state is more localised on one side of the core, CT occurs preferentially toward the branch attached on this side. Alternatively, the direction of the core-to-branch CT could be entirely determined by the fluctuations of the solvent field.

**Fig. 8:**
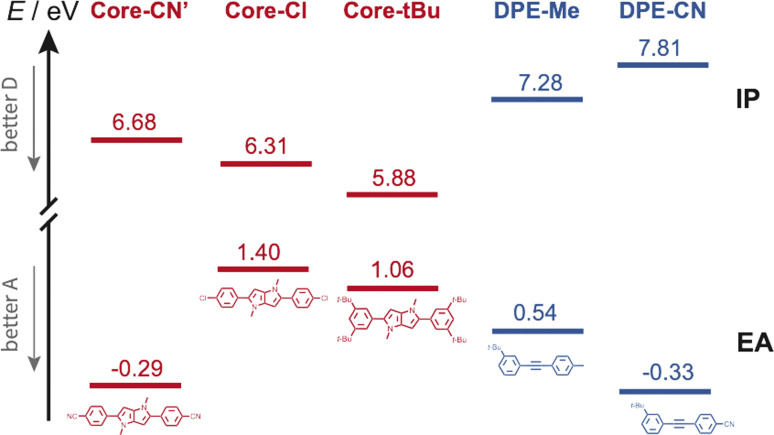
Ionisation energy (IE) and electron affinity (EA) of the various DHPP cores and DPE branches obtained from quantum-chemical calculations. Core-CN′ is the analogue of Core-CN shown in Fig. 1 without the phenyl groups on the N atoms.

Occurrence of this CT process depends on the nature of the terminal substituents on the core and branches, which determines their electron donating and withdrawing character. This dependence can be rationalised by considering the redox properties of the subunits, which are not available, or alternatively their ionisation energy (IE) and electron affinity (EA), which can be estimated from quantum-chemical calculations. As illustrated in Fig. 8, Core-CN is simultaneously a better acceptor and a better donor than DPE-Me. This accounts for the LE character of the lowest excited state of CN–Me. Replacement of the methyl on DPE by a cyano group makes DPE-CN a better acceptor than any of the cores. This agrees with the observation of the core-to-branch CT state with both CN–CN and *t*Bu-CN. The faster CT dynamics measured with *t*Bu-CN relative to CN–CN are consistent with the smaller IE of Core-*t*Bu compared to Core-CN, pointing to a larger driving force. Finally, Fig. 8 suggest that the smallest driving force for core-to-branch CT is for Cl–Me.

The lifetime of this CT state coincides with the fluorescence lifetime. Furthermore, the small radiative rate constants determined for CN–CN, *t*Bu-CN and Cl–Me (Table S3†) are consistent with CT emission. Consequently, this core-to-branch CT state should not be considered as a charge-separated state. The solvent dependence of the shape of the CT ESA band below 500 nm (Fig. 3B, S7 and S9†) and of the relative intensity of the –C≡C– and –C≡N bands (Fig. 4 bottom and S15†) suggests that the amount of CT increases with the polarity of the environment.

(3) Interbranch CT. The presence of two –C≡C– bands in the TRIR spectra of Cl–Me in highly polar media can only be explained by an excited state involving both DPE branches. The large frequency splitting implies that the electronic density on these two branches differs markedly. Therefore, an interbranch CT seems to be the most plausible explanation for these observations. The reason why this CT is operative with Cl–Me and not with CN–CN and *t*Bu-CN, probably originates from the relatively lower stability of the core-to-branch CT state mentioned above (Fig. 8). This interbranch CT state is also probably not a CS state, as excitation is most likely not entirely localised on the branches but should also affect the core. The reason why this interbranch CT state is only observed in highly polar environments is probably related to the solvation energy. Given the structure of these triads, the dipole moment of the interbranch CT state should be considerably larger than that of the core-to-branch CT state. Considering that the solvation energy associated with dipole–dipole interaction scales with the square of the solute dipole moment,^77^ the solvation energy of the interbranch CT state can be expected to be more than twice as large as that of the core-to-branch CT state. Consequently, the interbranch CT state could become more stable than the core-to-branch CT state when going from THF to BCN. This is consistent with the TRIR data, which reveal that excitation of Cl–Me is first followed by the population of the core-to-branch CT state, which then converts to the interbranch CT state on a timescale that is comparable with the diffusive motion of BCN.^78^ The changes in the relative intensity of the two –C≡C– bands observed when varying the composition of the THF/BCN mixture (Fig. S18†) suggests the presence of an equilibrium between these two CT states. As the BCN content increases, this equilibrium shifts progressively towards the interbranch CT state. Above a BCN content larger than 65%, the relative magnitude of the two bands remains constant, indicating that the equilibrium is totally on the side of the interbranch CT state.

### 3.2 The fate of the CT states

We now consider the decay of these CT states. All time-resolved fluorescence, electronic TA and TRIR measurements are congruent with a lifetime of both core-to-branch and interbranch CT states within ∼5 to 10 ns. No clear dependence of the lifetime on the nature of the substituents and on the solvent can be evidenced, except possibly for the shorter 2.1 ns lifetime of *t*Bu-CN in BCN. In principle, charge-recombination (CR) processes can be discussed in terms of Marcus ET theory.^79^ Generally, their dynamics accelerate upon decreasing the energy gap between the CT and neutral ground state, as expected for the inverted region.^1,80–83^ Such a dependence cannot be observed here. According to the electron donating and withdrawing strength of the core and branches discussed above (Fig. 8), the driving force for CR should decrease by going from Cl–Me to CN–CN and to *t*Bu-CN and upon increasing solvent polarity. However, both electronic and TRIR data reveal that, with the exception of *t*Bu-CN in BCN, the decay of the CT state occurs concurrently to the population of the triplet state localised on the DHPP core. Consequently, in those cases, CR populates the triplet state and not the ground state. In principle, triplet CR of a singlet CT state is spin forbidden. However, different mechanisms were shown to enable this process. The singlet and triplet states of a CS state with close to 100% CT character are quasi-degenerate and spin conversion is possible *via* hyperfine coupling.^84,85^ Otherwise, the presence of heavy atoms can generate sufficient spin–orbit coupling (SOC) to enable triplet recombination of CT states.^86–88^

Additionally, large SOC can be achieved if the orbitals associated with the electron and holes are not in the same plane and approach orthogonality. This mechanism, called spin–orbit charge transfer (SOCT) ISC, was shown to be operative for the triplet CR of exciplexes as well as CT states of non-planar D–A dyads.^89–92^ Considering the absence of heavy atom in the triads, the fact that the CT states are not charge-separated states with a negligibly small singlet-triplet gap, and the large angle between the DHPP core and the DPE branches, the triplet CR observed here most probably occurs *via* the SOCT-ISC mechanism.

According to TD-DFT calculations, up to seven triplet excited states are located below the LE state with an energy gap between this LE state and the T_1_ state as large as 1.2–1.3 eV. Therefore, triplet CR should be energetically favourable for Cl–Me and CN–CN in all solvents investigated. For *t*Bu-CN, no significant triplet population could be detected in BCN, suggesting that for this triad, the CT state is energetically too low for triplet recombination to compete with singlet CR to the ground state. The lower energy of the CT state should also accelerate singlet recombination, in agreement with the shorter lifetime measured experimentally. In THF, the triplet yield is smaller than for the other triads, indicating that both CR pathways are probably operative.

## 4 Conclusions

This investigation illustrates how the excited-state properties, including the location of the excitation, of formally centrosymmetric conjugated molecules can be controlled by fine tuning structural and environmental parameters. Three different types of symmetry-broken CT states could be evidenced, depending on the substituents and solvent. The first involves a transition from a purely quadrupolar to a dipolar state, through the redistribution of the excitation within the DHPP core. This intracore CT occurs when two strongly electron-withdrawing groups are located at positions 2 and 5 of the DHPP core and electron-neutral substituents are present at positions 1 and 4. This process is mostly driven by the asymmetry of the solvent field and the extent of symmetry breaking increases with the solvent polarity. The second involves a transfer of charge from the core to one of the two equivalent DPE branches. It takes place with electron-withdrawing peripheral substituents. In this case, it is not clear whether the origin of the symmetry breaking is the solvent field as well or the initial symmetry breaking of the local excitation in the core. Transient two-dimensional IR experiments are planned to get deeper insight into this question. Finally, the third CT process is somehow related to the second one and involves an increased localisation of the positive charge on the other DPE branch, leading to a state similar to that resulting from a SB-CS between two identical molecules. It is observed in the absence of any particular electron-donating or -withdrawing groups. Such a control of the nature of CT states through small structural modifications and proper selection of the environment could in principle be applied to larger molecular architectures and could be advantageously exploited for various applications, such as sensing and optoelectronics.

## Data availability

All data can be downloaded from https://doi.org/10.26037/yareta:zloeqsw67faw5lfhtfgrrmmpfu.

## Author contributions

C. Govind and E. Balanikas designed and performed the spectroscopic experiments, analysed the data and contributed to the initial draft. D. T. Gryko supervised the synthesis and characterisation all fours compounds, that was carried out by G. Sanil. E. Vauthey supervised the study, performed the QM calculations and wrote the final version with the help of all authors.

## Conflicts of interest

There are no conflicts to declare.

## Supplementary Material

SC-OLF-D4SC05419A-s001
