# Supplementary material for: Structural and solvent modulation of symmetry-breaking charge-transfer pathways in molecular triads
Source: Chem Sci. 2024 Sep 26;15(42):17362–71. doi: 10.1039/d4sc05419a (PMC11445701; doi:10.1039/d4sc05419a)
Supplement: SC-015-D4SC05419A-s001 [file SC-015-D4SC05419A-s001.pdf]

Supplementary Information (SI) for Chemical Science.
This journal is © The Royal Society of Chemistry 2024

Structural and solvent modulation of symmetry-breaking charge-transfer pathways in molecular triads

Chinju Govind[a], Evangelos Balanikas[a], Gana Sanil[b], Daniel T. Gryko[b], and Eric Vauthey*[a]

[a]*Department of Physical Chemistry, University of Geneva, 30 Quai Ernest-Ansermet, CH-1211 Geneva 4, Switzerland. E-mail: eric.vauthey@unige.ch*

[b] *Institute of Organic Chemistry, Polish Academy of Sciences, 01-224 Warsaw, Poland*

Contents

S1 Experimental 5

S1.1 Samples . . . 5

S1.2 Stationary spectroscopy . . . 5

S1.3 Time-resolved fluorescence . . . 5

S1.4 Electronic transient absorption spectroscopy . . . 5

S1.5 Time-resolved IR spectroscopy . . . 5

S2 Quantum-chemical calculations 7

S3 Nanosecond time-resolved fluorescence 10

S4 Electronic transient absorption spectroscopy 11

S5 Time-resolved IR spectroscopy 15

List of Figures

S1 Energy of the analogue of **CN-CN** without the *t*-Bu groups as a fonction of the dihedral angle depicted on the top left panel calculated at the DFT level. The dashed line is the thermal energy at room temperature. 7

S2 Energy, oscillator strength, dominant contributions to the lowest electronic transitions of the triads and relevant molecular orbitals obtained from TD-DFT calculations. 8

S3 Molecular orbitals associated with the hole of the radical cation (β-LUMO) and the unpaired electron of the radical anion (α-HOMO) of **CN-CN** obtained from unrestricted calculations (CAM-B3LYP/6-31g(d,p)). 9

S4 Nanosecond fluorescence dynamics measured with the triads in THF (except toluene for **tBu-CN**), instrument response function and best exponential fit. **CN-Me** and **CN-CN** were excited at 395 nm; **tBu-CN** and **Cl-Me** were excited at 375 nm. The detection wavelength is given between parantheses. 10

S5 Electronic transient absorption spectra recorded at various time delays after 400 nm (<2 ns) or 355 nm excitation (>2 ns) of **CN-Me** in toluene (left) and benzonitrile (right), together with the negative stationary absorption and stimulated emission spectra (grey shading). 11

S6 Evolution-associated difference absorption spectra and time constants obtained from a global analysis of the TA data measured with **CN-Me** in different solvents assuming a series of successive exponential steps (A→B→ ...). 11

S7 Electronic transient absorption spectra recorded at various time delays after 400 nm (<2 ns) or 355 nm excitation (>2 ns) of **CN-CN** in toluene (left) and benzonitrile (right), together with the negative stationary absorption and stimulated emission spectra (grey shading). 12

S8 Evolution-associated difference absorption spectra and time constants obtained from a global analysis of the TA data measured with **CN-CN** in different solvents assuming a series of successive exponential steps (A→B→ ...). 12

S9 Electronic transient absorption spectra recorded at various time delays after 360 nm (<2 ns) or 355 nm excitation (>2 ns) of **tBu-CN** in toluene (left), tetrahydrofuran (middle) and benzonitrile (right), together with the negative stationary absorption and stimulated emission spectra (grey shading). 13

S10 Evolution-associated difference absorption spectra and time constants obtained from a global analysis of the TA data measured with **tBu-CN** in different solvents assuming a series of successive exponential steps (A→B→ ...). 13

S11 Electronic transient absorption spectra recorded at various time delays after 360 nm (<2 ns) or 355 nm excitation (>2 ns) of **Cl-Me** in toluene (left), tetrahydrofuran (middle) and benzonitrile (right), together with the negative stationary absorption and stimulated emission spectra (grey shading). 14

S12 Evolution-associated difference absorption spectra and time constants obtained from a global analysis of the TA data measured with **Cl-Me** in different solvents assuming a series of successive exponential steps (A→B→ ...). 14

S13 Evolution-associated difference absorption spectra and time constants obtained from a global analysis of the TRIR data measured with **CN-Me** in different solvents assuming a series of successive exponential steps (A→B→ ...). 15

S14 Evolution-associated difference absorption spectra and time constants obtained from a global analysis of the TRIR data measured with **CN-CN** in different solvents assuming a series of successive exponential steps (A→B→ ...). 15

S15 Time-resolved IR spectra recorded at various delays after 360 nm excitation of **tBu-CN** in toluene (lelft), tetrahydrofuran (middle) and benzonitrile (right). 15

S16 Evolution-associated difference absorption spectra and time constants obtained from a global analysis of the TRIR data measured with **tBu-CN** in different solvents assuming a series of successive exponential steps (A→B→ ...). 16

S17 Evolution-associated difference absorption spectra and time constants obtained from a global analysis of the TRIR data measured with **Cl-Me** in pure solvents assuming a series of successive exponential steps (A→B→ ...). 16
S18 Time-resolved IR spectra recorded at various delays after 360 nm excitation of **Cl-Me** in tetrahydrofuran/benzonitrile mixtures. 16
S19 Evolution-associated difference absorption spectra and time constants obtained from a global analysis of the TRIR data measured with **Cl-Me** in tetrahydrofuran/benzonitrile mixtures assuming a series of successive exponential steps (A→B→ ...). 16
S20 Time dependence of the polarisation anisotropy of the -C≡N ESA band measured after 400 nm excitation of **Core-CN** in various solvents and best exponential fits. 17
S21 Time dependence of the polarisation anisotropy of the -C≡N ESA band measured after 400 nm excitation of **CN-Me** in various solvents and best exponential fits. 17
S22 Time dependence of the polarisation anisotropy of the -C≡N ESA band measured after 400 nm excitation of **CN-CN** in toluene and best bi-exponential fit. 17

List of Tables

S1 Frequencies and intensities (between brackets, in km/mol) of the -C≡C- and -C≡N IR bands of **CN-CN** in the ground state and of its radical cation and anion calculated at the DFT level (CAM-B3LYP/6-31g(d,p)). No correction factor was applied to the frequencies. 9
S2 Frequencies and intensities (between brackets, in km/mol) of the -C≡C- IR bands of **Cl-Me** in the ground state and of its radical cation and anion calculated at the DFT level (CAM-B3LYP/6-61g(d,p)). No correction factor was applied to the frequencies. 9
S3 Fluorescence quantum yields, fluorescence lifetimes and radiative rate constants in THF. 10

S1 Experimental

S1.1 Samples

The synthesis of all four triads was reported in ref. 1. All solvents were of the highest purity commercially available and were used as received.

S1.2 Stationary spectroscopy

Electronic absorption spectra were measured using a Cary 50 spectrometer, whereas emission spectra were recorded on a Horiba FluoroMax-4 spectrofluorometer. The fluorescence spectra were corrected using a set secondary emissive standards.[2]

The stimulated emission spectra were calculated by mutiplying the spontaneous fluorescence intensity by λ^4, where λ is the wavelength.[3]

S1.3 Time-resolved fluorescence

Nanosecond time-resolved fluorescence dynamics were measured using the time-correlated single photon counting (TCSPC) technique with a setup similar to that described in Ref. 4. Excitation was carried out with a laser diodes at 395 nm and 375 nm (LDH-P-C-400B and (LDH-P-C-375 PicoQuant). The pulse duration was 60 ps, and the full width at half maximum (FWHM) of the instrument response function (IRF) was about 200 ps. A Glan–Taylor polarizer was placed in front of the sample to ensure linearly polarized excitation light. The fluorescence was collected at the magic angle relative to the polarization of the excitation pulse and passed through an interference filter.

S1.4 Electronic transient absorption spectroscopy

Transient absorption (TA) measurements on the fs-ps timescales were carried out with the same setup as described in ref. 5 with the exception that the delay stage was moved to the probe instead of the pump path. Briefly, the output pulses of a regeneratively amplified Ti:Sapphire system (Spectra-Physics, Solstice, 800 nm, 35 fs, 5 KHz) were split into pump and probe pulses and were mechanically chopped down to 0.5 kHz and 1 kHz respectively. Excitation of **CN-Me** and **CN-CN** was performed at 400 nm. These pulses were generated by frequency doubling 800 nm pulses. Excitation of **tBu-CN** and **Cl-CN** was done at 360 nm with pulses generated upon frequency doubling the 720 nm output of an optical parametric amplifier (TOPAS-Prime combined with a NirUVis module, Light Conversion). For probing, a white light supercontinuum (320 - 750 nm), generated by focusing a fraction of the Solstice output into a 3 mm CaF_2 plate, was used. The pump and probe pulses were separated in time using a mechanical delay stage (Physics Instruments) allowing for a pump-probe delay up to 1.8 ns. The polarisation of the pump pulses was at magic angle with respect to the probe pulses using a set of half wave plates and polarisers. Transient absorption spectra were detected using reference detection with two spectrographs equipped with CCDs (Entwicklungsbüro Stresing, Berlin). The pump pulses were focused to an approximately 300 μm^2 spot on the sample. The pump fluence at the sample position was attenuated to about 0.3 mJ/cm^2. The liquid samples were located in a 1 mm pathlength cell under constant nitrogen bubbling. The FWHM of the IRF was determined to be around 100 - 400 fs depending on the probe wavelength by optical Kerr effect measurements in the solvent used for the TA measurement as described in ref. 6.

TA measurements on the ns-μs timescales were performed with a setup described in detail in ref. 7. Excitation was performed at 355 nm using a passively Q-switched, frequency tripled Nd:YAG laser (Teem Photonics, Powerchip NanoUV) producing 300 ps pulses at 500 Hz. The pump fluence on the sample was around 2.5 mJ/cm^2. Probing was achieved as in the fs-ps transient absorption experiment. The FWHM of the IRF was around 370 ps.

S1.5 Time-resolved IR spectroscopy

Femtosecond time-resolved IR (TRIR) spectra were obtained using a homebuilt setup based on a Ti:Sapphire amplified system (Spectra Physics Solstice) producing 100 fs pulses at 800 nm and 1 kHz repetition rate as described in detail previously.[8,9] excitation was carried out either at 400 nm (**CN-Me** and **CN-CN**) by frequency doubling a fraction of the amplifier output or at 360 nm (**tBu-CN** and **Cl-Me**) using a TOPAS-Prime combined

with a NirUVis module (Light Conversion). The linearity of the signal amplitude with respect to pump intensity was checked before each experiment and proper adjustment was made to ensure the maximum signal in a linear regime. The polarization was controlled with a combination of Glan-Laser polariser and zero-order half-wave plate, limiting the time resolution of the experiment to 300 fs. The pulses were focused on the sample onto 350 μm spot, resulting in a fluence of 0.05-0.3 mJ/cm^2. Mid-IR probe pulses at around 4.7-5.2 μm where generated by difference frequency mixing of the output of an optical parametric amplifier (Light Conversion, TOPAS-C with NDFG module) that was pumped at 800 nm. The polarization of the IR beam was controlled using a wire-grid polarizer. Two horizontally polarized IR beams were produced with a CaF2 wedge and focused onto the sample. One of the beams was overlapped with the pump beam, whereas the second was used as reference. Both IR beams were focused onto the entrance slit of an imaging spectrograph (Horiba, Triax 190, 150 lines/mm) equipped with a liquid nitrogen cooled 2 x 64 element MCT array (Infrared Systems Development), giving a resolution of 3-4 cm^{-1} in the -C≡C- stretching region. The sample area and the detection system were placed in a box that was purged with water- and carbon dioxide-free air for at least one hour before each experiment. The average of 500 signal shots was taken to collect one data point with the polarization of the pump pulses at the magic angle with respect to that of the IR pulse. This procedure was carried out for at least four times depending on the signal reproducibility and intensity. For anisotropy measurements, the polarization of the pump beam either parallel or perpendicular to the probe polarization.

S2 Quantum-chemical calculations

All calculations were carried out in the gas phase at the density functional theory (DFT) or time-dependent (TD) DFT levels using the CAM-B3LYP functional,[10] and the 6-31g(d,p) basis set. All calculations were performed using Gaussian16 (Rev. B).[11] To speed up the calculations, the *t*-Bu groups in **CN-Me**, **CN-Me** and **Cl-Me** were replaced by hydrogen atoms.

The ionisation energies were calculated by taking the energy difference between the radical cations and the neutrals at the equilibrium geometry of the neutral ground state. The electron affinities were calculated likewise but using the energy of the radical anions at the neutral ground state geometry.

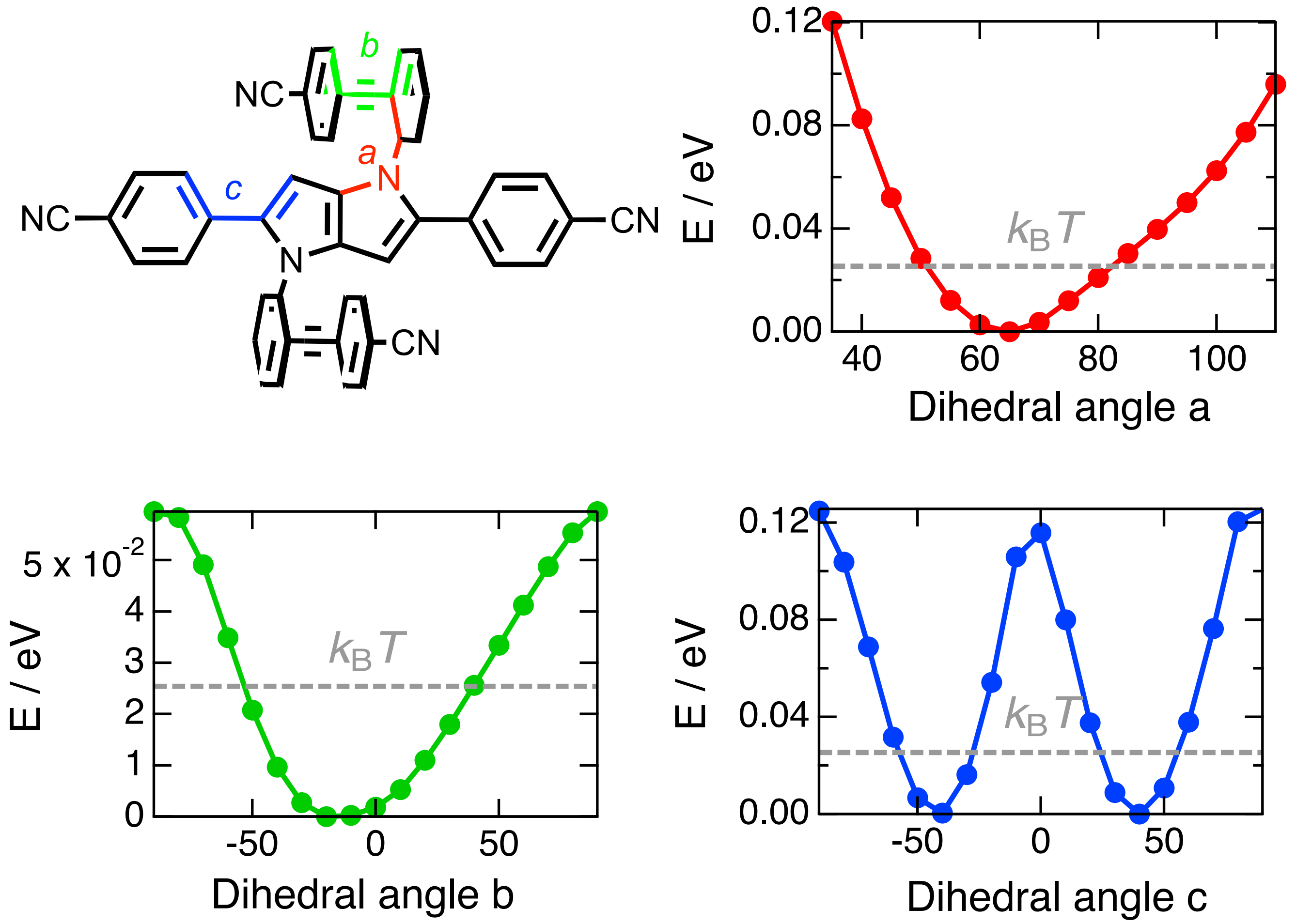

Figure S1 Energy of the analogue of **CN-CN** without the *t*-Bu groups as a fonction of the dihedral angle depicted on the top left panel calculated at the DFT level. The dashed line is the thermal energy at room temperature.

	CN-Me	CN-CN	tBu-CN	Cl-Me
$S_3 \leftarrow S_0$			E=4.03 eV, f=1.0<br>HOMO→LUMO+2	
$S_2 \leftarrow S_0$	E=4.1 eV, f=0<br>HOMO→LUMO+1	E=3.77 eV, f=0<br>HOMO→LUMO	E=3.46 eV, f=0<br>HOMO→LUMO+1	E=3.97 eV, f=0<br>HOMO→LUMO+1
$S_1 \leftarrow S_0$	E=3.65 eV, f=1.1<br>HOMO→LUMO	E=3.65 eV, f=0.74<br>HOMO→LUMO+1<br>HOMO→LUMO+2	E=3.45 eV, f=0<br>HOMO→LUMO	E=3.86 eV, f=0.5<br>HOMO→LUMO<br>HOMO→LUMO+2

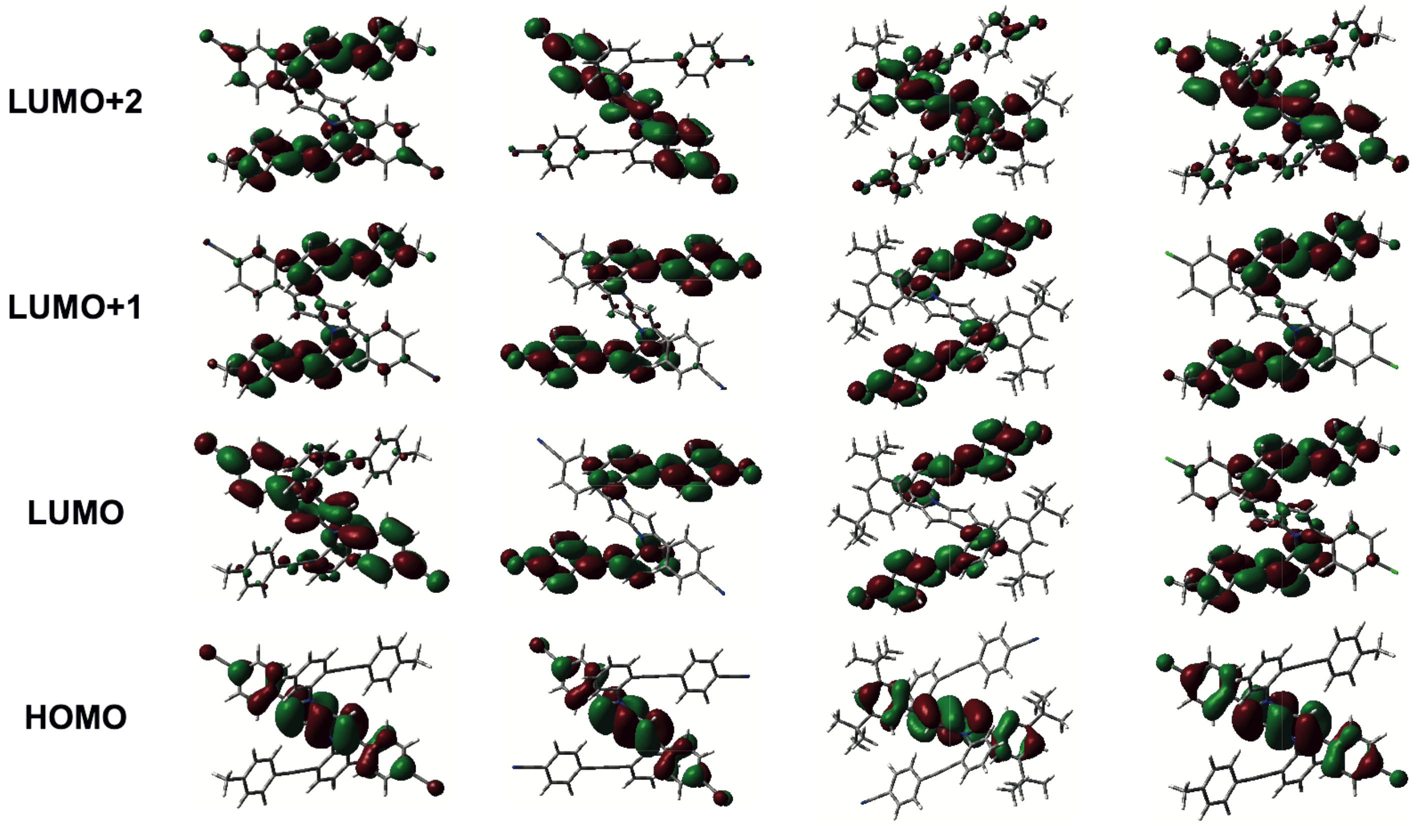

Figure S2 Energy, oscillator strength, dominant contributions to the lowest electronic transitions of the triads and relevant molecular orbitals obtained from TD-DFT calculations.

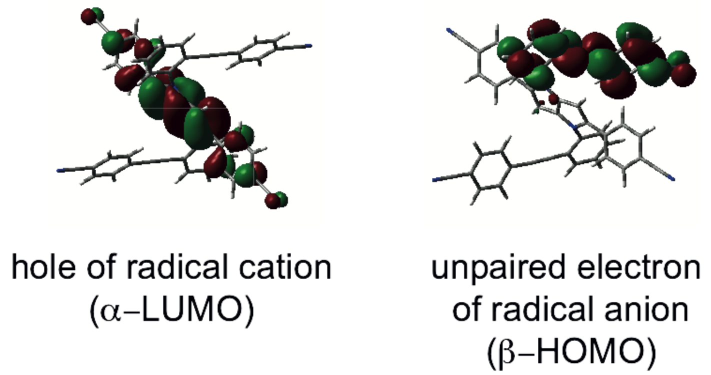

Figure S3 Molecular orbitals associated with the hole of the radical cation (β-LUMO) and the unpaired electron of the radical anion (α-HOMO) of **CN-CN** obtained from unrestricted calculations (CAM-B3LYP/6-31g(d,p)).

Table S1 Frequencies and intensities (between brackets, in km/mol) of the -C≡C- and -C≡N IR bands of **CN-CN** in the ground state and of its radical cation and anion calculated at the DFT level (CAM-B3LYP/6-31g(d,p)). No correction factor was applied to the frequencies.

	-C≡C- (s)	-C≡C- (a)	core -C≡N (a)	core -C≡N (s)	branch -C≡N (a)	branch -C≡N (s)
Neutral	2380 (0)	2380 (20)	2409 (150)	2410 (0)	2412 (93)	2412 (0)
Cation	2375 (0)	2375 (60)	2418 (1)	2418 (0)	2417 (40)	2417 (0)
Anion	2378 (72)	2342 (980)	2400 (210)	2401 (105)	2326 (1315)	2407 (70)

Table S2 Frequencies and intensities (between brackets, in km/mol) of the -C≡C- IR bands of **Cl-Me** in the ground state and of its radical cation and anion calculated at the DFT level (CAM-B3LYP/6-61g(d,p)). No correction factor was applied to the frequencies.

	-C≡C- (s)	-C≡C- (a)
Neutral	2379 (0)	2379 (27)
Cation	2371 (0)	2371 (300))
Anion	2380 (0.5)	2238 (370)

S3 Nanosecond time-resolved fluorescence

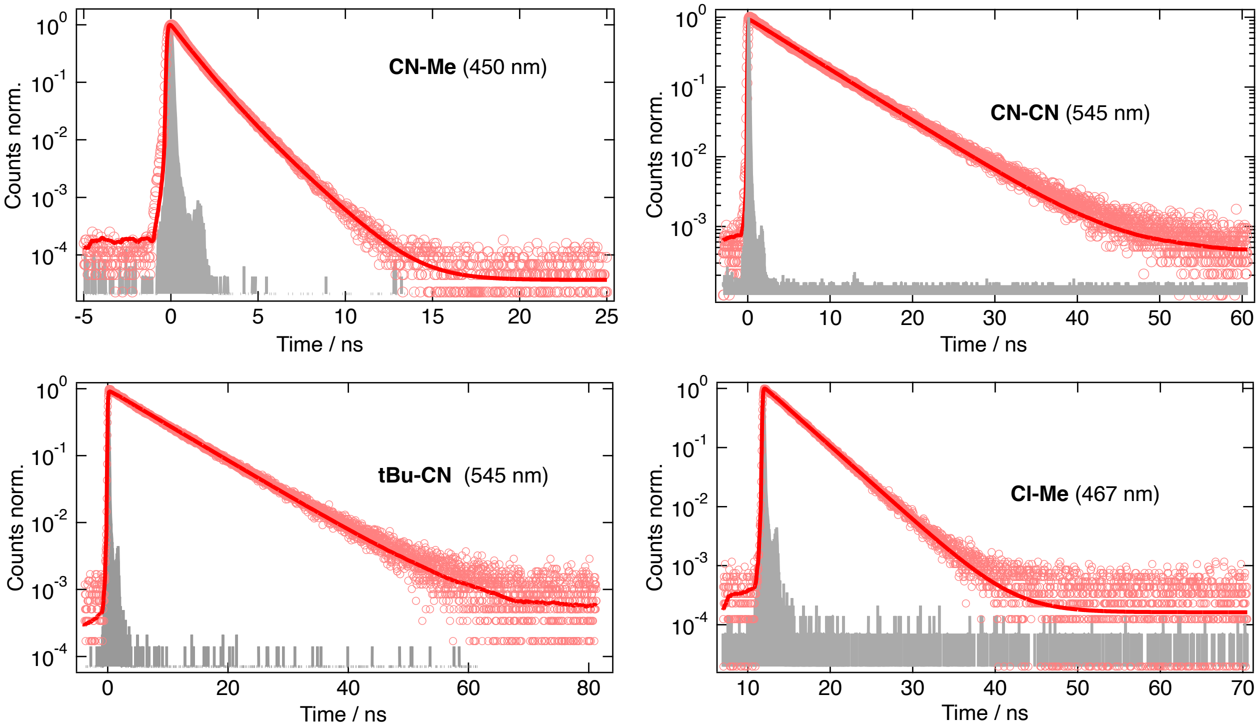

Figure S4 Nanosecond fluorescence dynamics measured with the triads in THF (except toluene for **tBu-CN**), instrument response function and best exponential fit. **CN-Me** and **CN-CN** were excited at 395 nm; **tBu-CN** and **Cl-Me** were excited at 375 nm. The detection wavelength is given between parantheses.

Table S3 Fluorescence quantum yields, fluorescence lifetimes and radiative rate constants in THF.

molecule	Φ_{fl}^{a}	τ_{fl} / ns	k_{rad}/ 10^8 s^{-1}
**Core-CN**[b]	0.75	1.35	5.6
**CN-Me**	0.87	1.14	7.6
**CN-CN**	0.02	5.9	0.03
**tBu-CN**[c]	0.06	8.2	0.07
**Cl-Me**	0.03	3.5	0.08

[a] from ref. 1; [b] from ref. 12; [c] in toluene.

S4 Electronic transient absorption spectroscopy

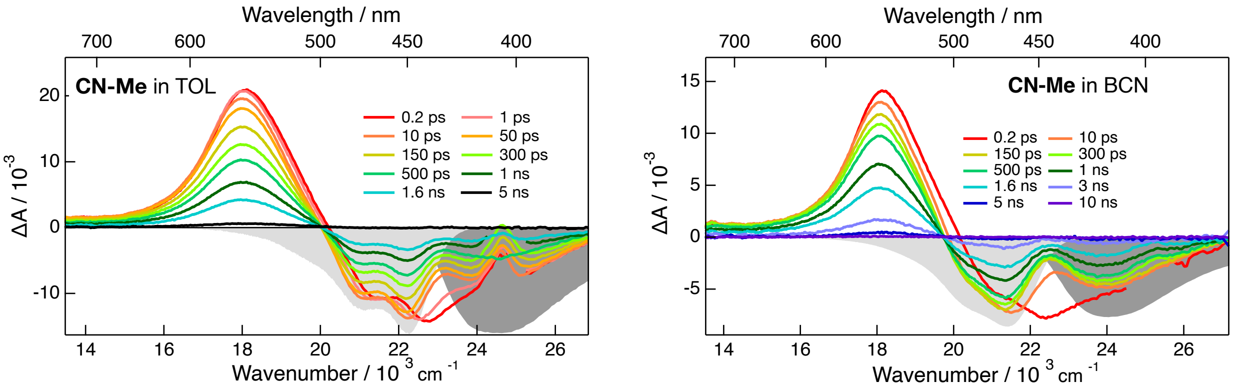

Figure S5 Electronic transient absorption spectra recorded at various time delays after 400 nm (<2 ns) or 355 nm excitation (>2 ns) of **CN-Me** in toluene (left) and benzonitrile (right), together with the negative stationary absorption and stimulated emission spectra (grey shading).

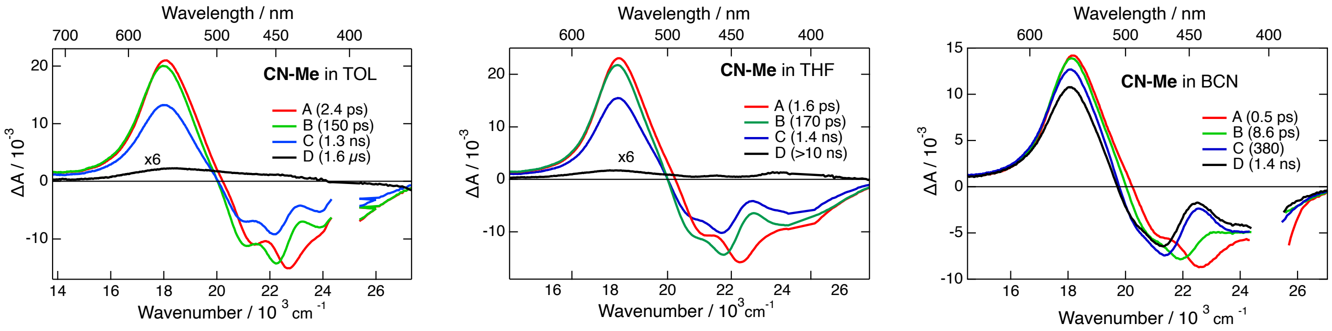

Figure S6 Evolution-associated difference absorption spectra and time constants obtained from a global analysis of the TA data measured with **CN-Me** in different solvents assuming a series of successive exponential steps (A→B→ . . .).

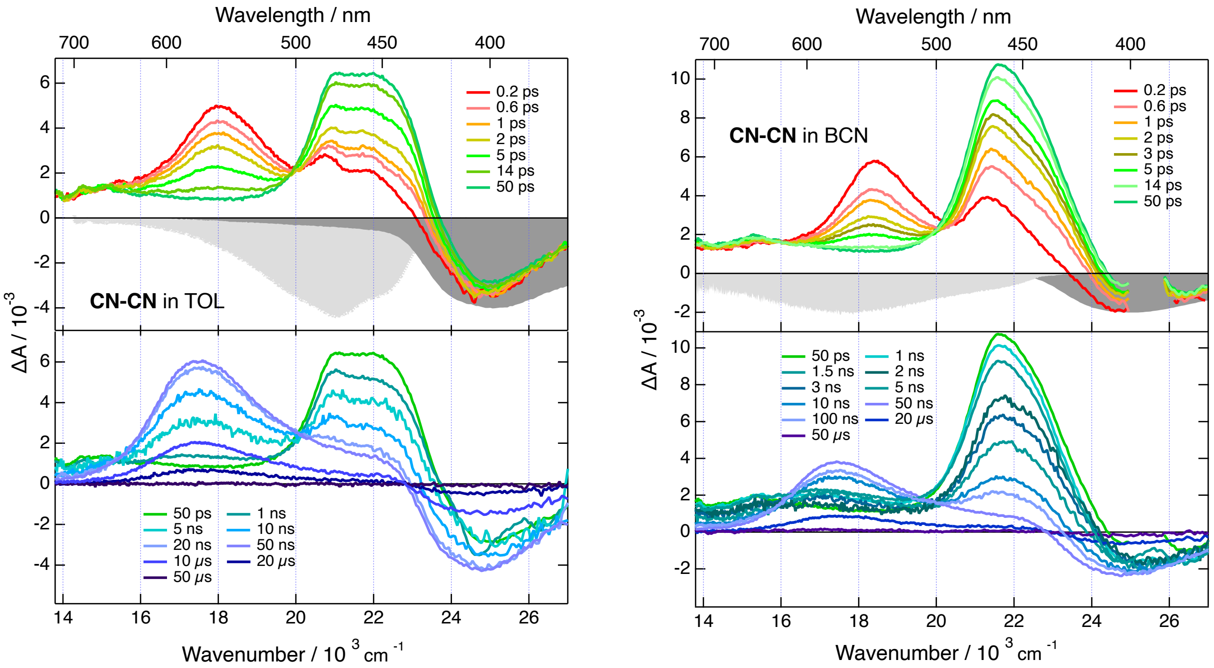

Figure S7 Electronic transient absorption spectra recorded at various time delays after 400 nm (<2 ns) or 355 nm excitation (>2 ns) of **CN-CN** in toluene (left) and benzonitrile (right), together with the negative stationary absorption and stimulated emission spectra (grey shading).

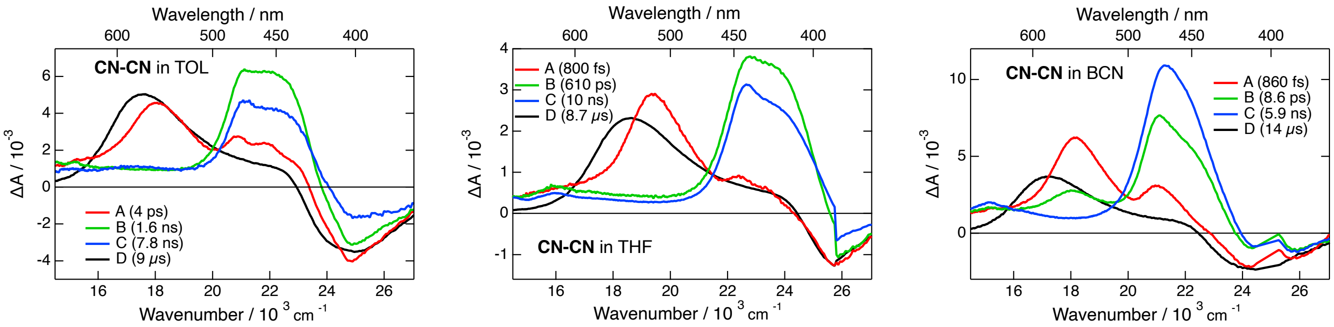

Figure S8 Evolution-associated difference absorption spectra and time constants obtained from a global analysis of the TA data measured with **CN-CN** in different solvents assuming a series of successive exponential steps (A→B→ . . .).

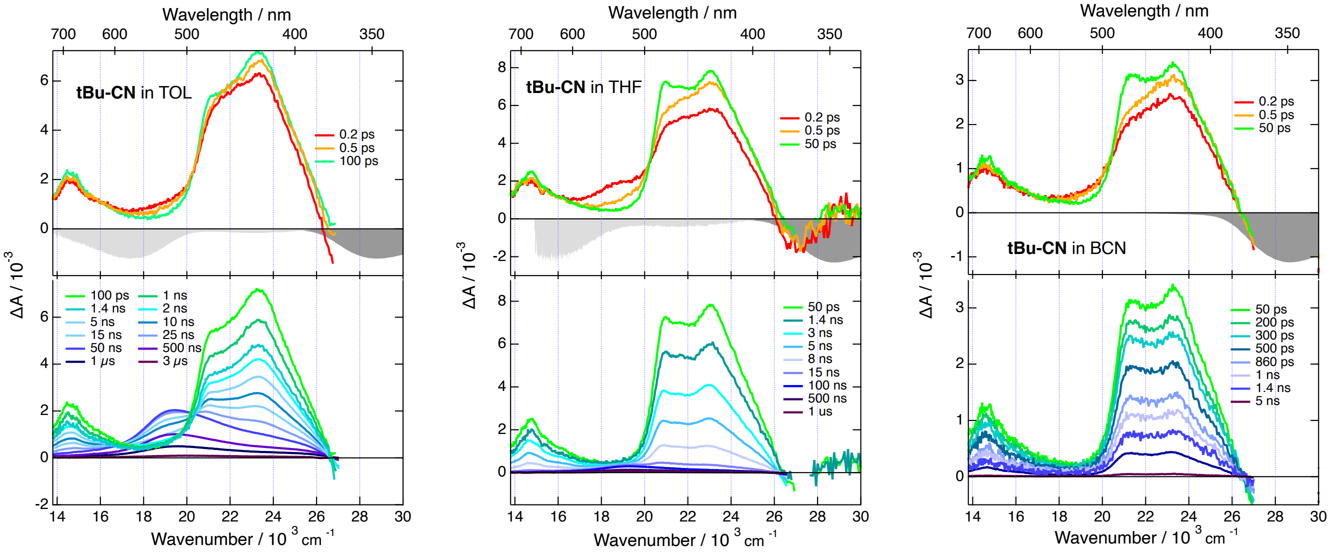

Figure S9 Electronic transient absorption spectra recorded at various time delays after 360 nm (<2 ns) or 355 nm excitation (>2 ns) of **tBu-CN** in toluene (left), tetrahydrofuran (middle) and benzonitrile (right), together with the negative stationary absorption and stimulated emission spectra (grey shading).

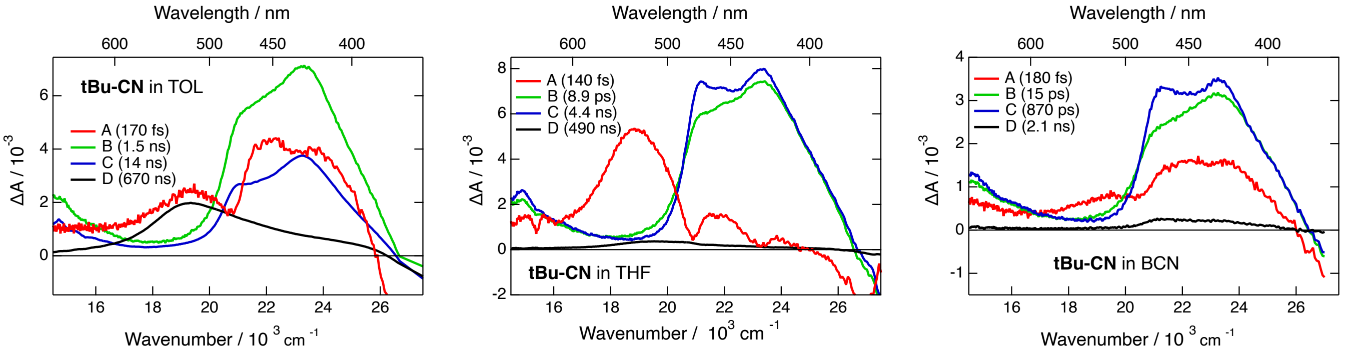

Figure S10 Evolution-associated difference absorption spectra and time constants obtained from a global analysis of the TA data measured with **tBu-CN** in different solvents assuming a series of successive exponential steps (A→B→ ...).

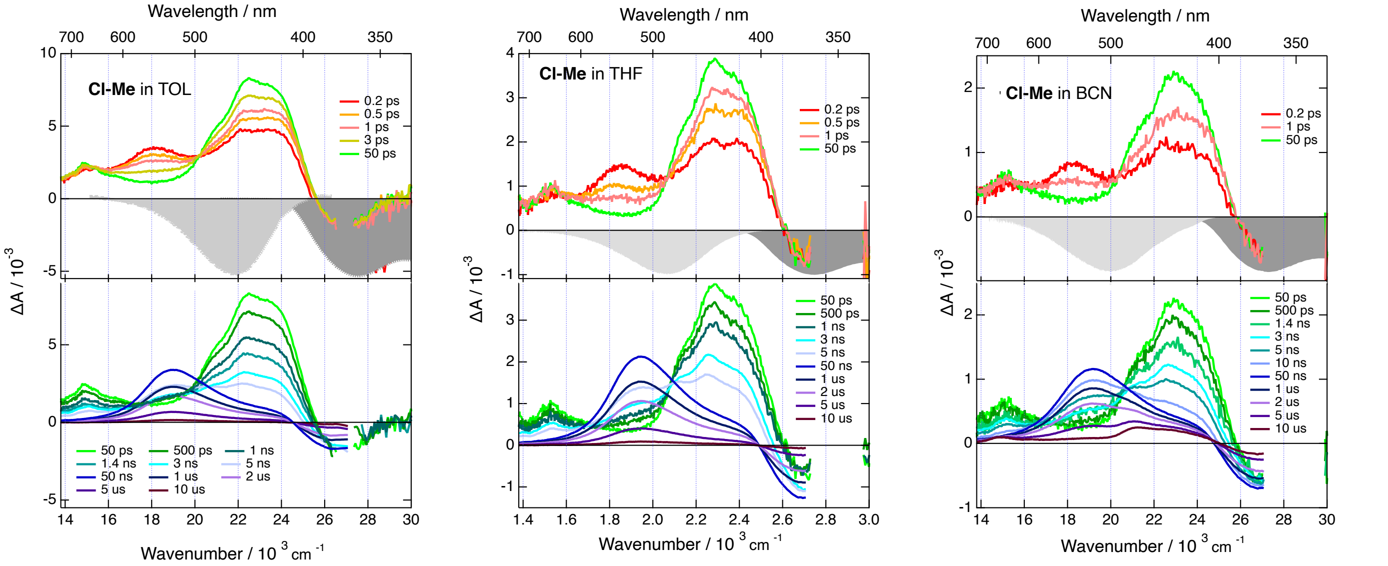

Figure S11 Electronic transient absorption spectra recorded at various time delays after 360 nm (<2 ns) or 355 nm excitation (>2 ns) of **Cl-Me** in toluene (left), tetrahydrofuran (middle) and benzonitrile (right), together with the negative stationary absorption and stimulated emission spectra (grey shading).

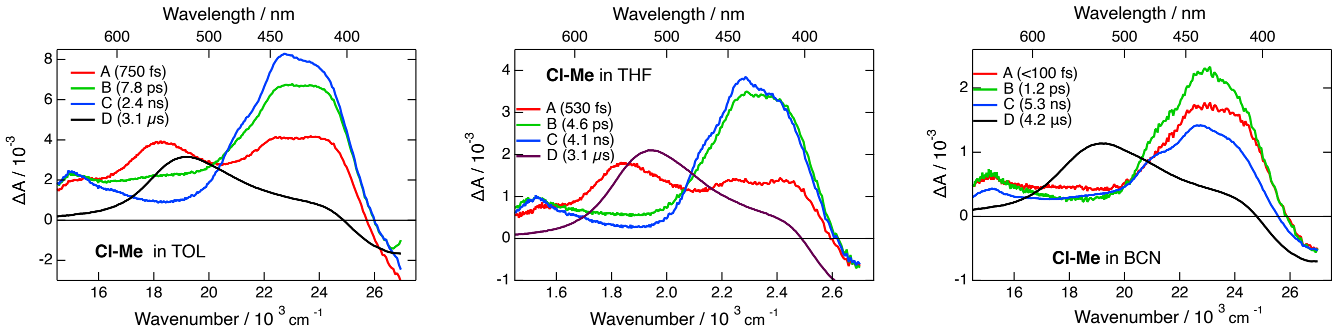

Figure S12 Evolution-associated difference absorption spectra and time constants obtained from a global analysis of the TA data measured with **Cl-Me** in different solvents assuming a series of successive exponential steps (A→B→ . . .).

S5 Time-resolved IR spectroscopy

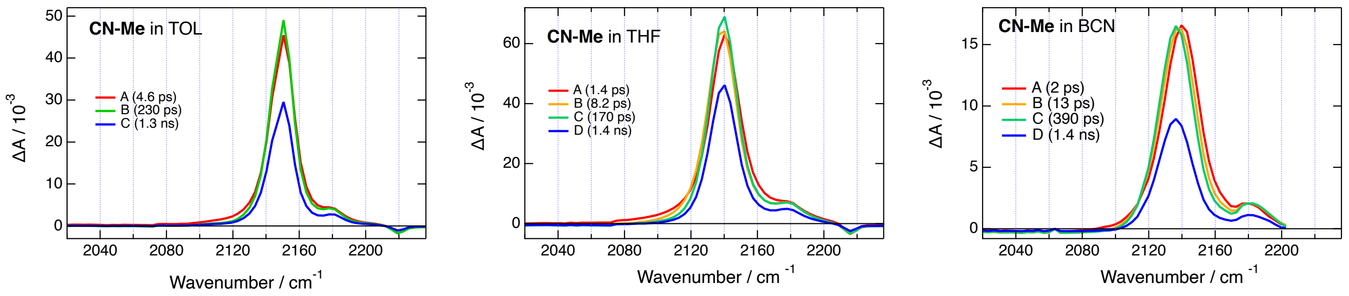

Figure S13 Evolution-associated difference absorption spectra and time constants obtained from a global analysis of the TRIR data measured with **CN-Me** in different solvents assuming a series of successive exponential steps (A→B→ ...).

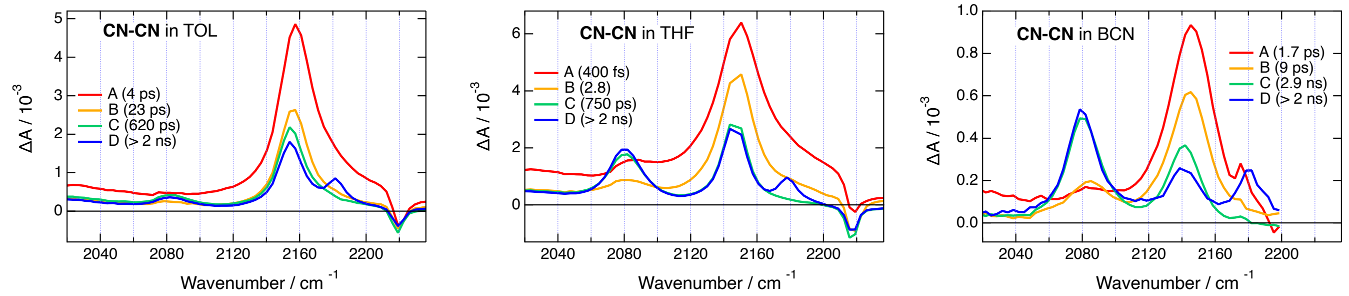

Figure S14 Evolution-associated difference absorption spectra and time constants obtained from a global analysis of the TRIR data measured with **CN-CN** in different solvents assuming a series of successive exponential steps (A→B→ ...).

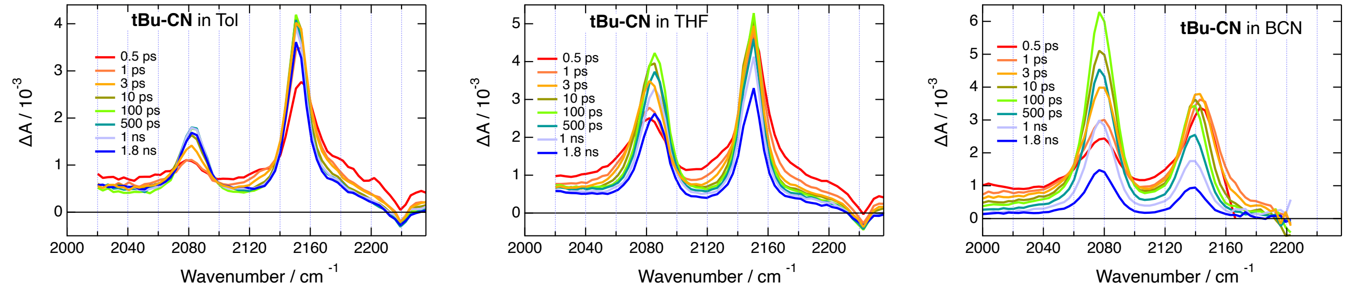

Figure S15 Time-resolved IR spectra recorded at various delays after 360 nm excitation of **tBu-CN** in toluene (lelft), tetrahydrofuran (middle) and benzonitrile (right).

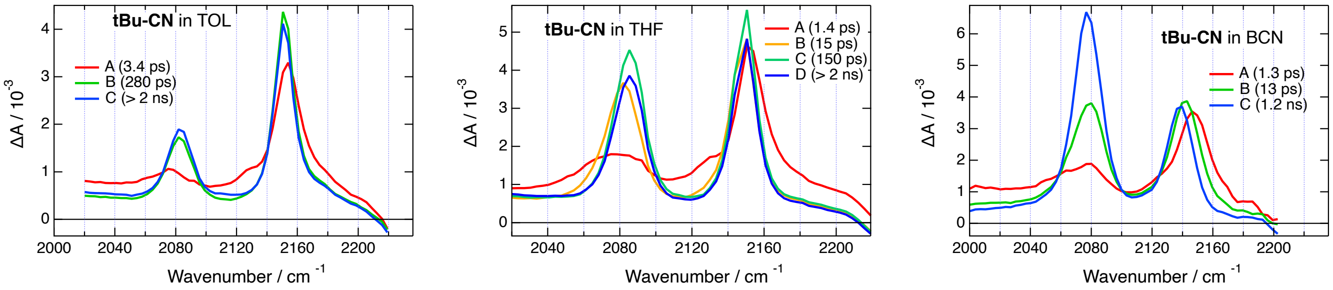

Figure S16 Evolution-associated difference absorption spectra and time constants obtained from a global analysis of the TRIR data measured with **tBu-CN** in different solvents assuming a series of successive exponential steps (A→B→ . . .).

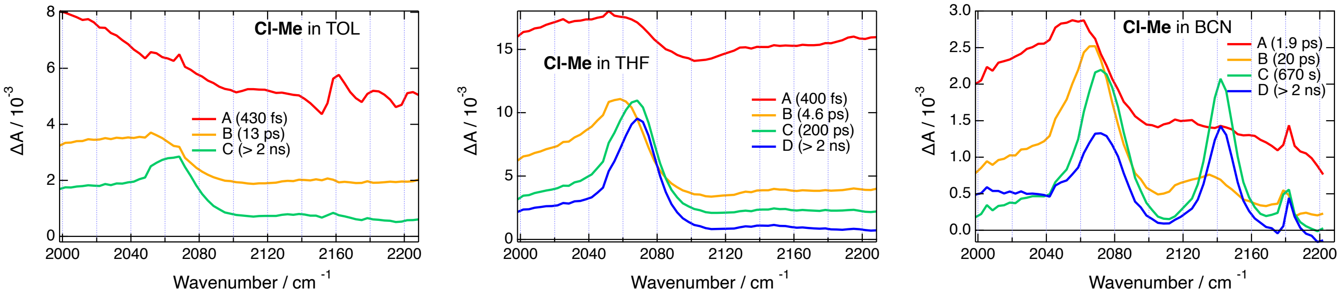

Figure S17 Evolution-associated difference absorption spectra and time constants obtained from a global analysis of the TRIR data measured with **Cl-Me** in pure solvents assuming a series of successive exponential steps (A→B→ . . .).

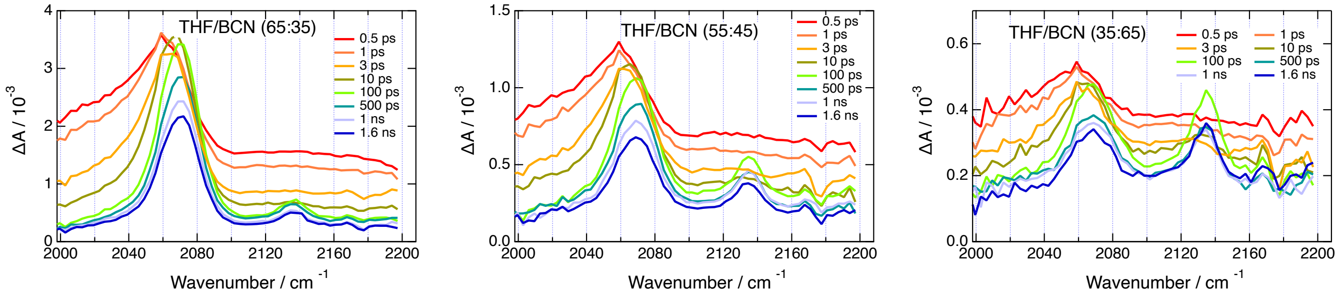

Figure S18 Time-resolved IR spectra recorded at various delays after 360 nm excitation of **Cl-Me** in tetrahydrofuran/benzonitrile mixtures.

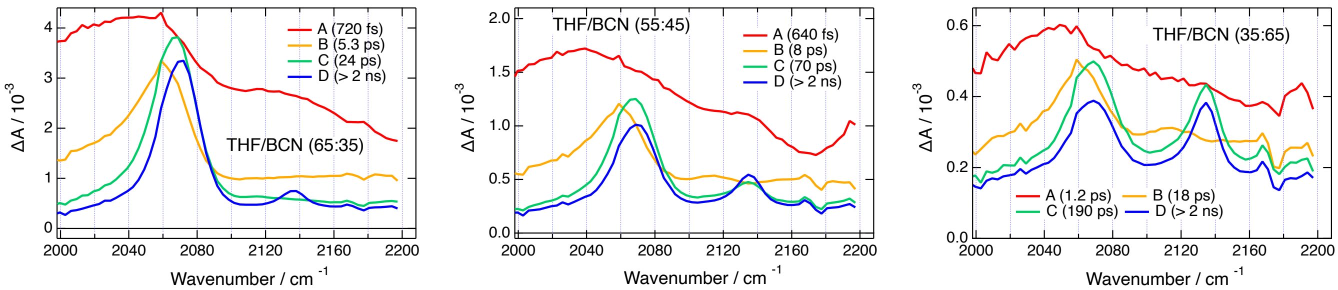

Figure S19 Evolution-associated difference absorption spectra and time constants obtained from a global analysis of the TRIR data measured with **Cl-Me** in tetrahydrofuran/benzonitrile mixtures assuming a series of successive exponential steps (A→B→ . . .).

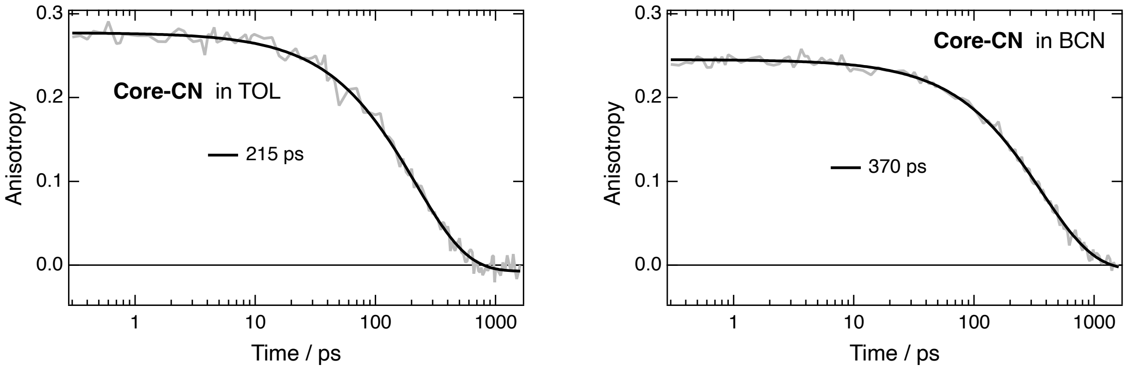

Figure S20 Time dependence of the polarisation anisotropy of the -C≡N ESA band measured after 400 nm excitation of **Core-CN** in various solvents and best exponential fits.

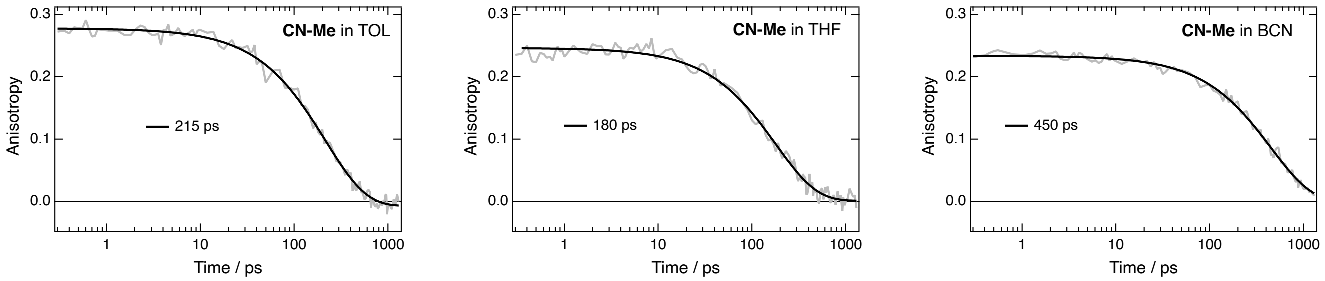

Figure S21 Time dependence of the polarisation anisotropy of the -C≡N ESA band measured after 400 nm excitation of **CN-Me** in various solvents and best exponential fits.

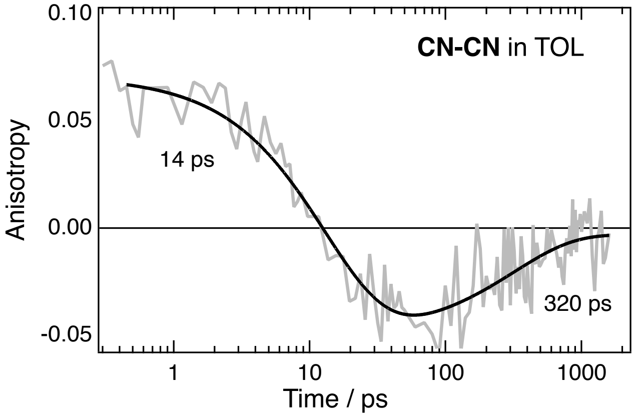

Figure S22 Time dependence of the polarisation anisotropy of the -C≡N ESA band measured after 400 nm excitation of **CN-CN** in toluene and best bi-exponential fit.

References

[1] G. Sanil, M. Krzeszewski, W. Chaładaj, W. Danikiewicz, I. Knysh, Ł. Dobrzycki, O. Staszewska-Krajewska, M. K. Cyrański, D. Jacquemin and D. T. Gryko, *Angew. Chem. Int. Ed.*, 2023, **62**, e202311123.

[2] J. A. Gardecki and M. Maroncelli, *Appl. Spectrosc.*, 1998, **52**, 1179–1189.

[3] A. V. Deshpande, A. Beidoun, A. Penzkofer and G. Wagenblast, *Chem. Phys.*, 1990, **142**, 123–131.

[4] P.-A. Muller, C. Högemann, X. Allonas, P. Jacques and E. Vauthey, *Chem. Phys. Lett.*, 2000, **326**, 321–327.

[5] J. S. Beckwith, A. Aster and E. Vauthey, *Phys. Chem. Chem. Phys.*, 2022, **24**, 568–577.

[6] J. S. Beckwith, C. A. Rumble and E. Vauthey, *Int. Rev. Phys. Chem.*, 2020, **39**, 135–216.

[7] B. Lang, S. Mosquera-Vazquez, D. Lovy, P. Sherin, V. Markovic and E. Vauthey, *Rev. Sci. Instrum.*, 2013, **84**, 073107–8.

[8] M. Koch, R. Letrun and E. Vauthey, *J. Am. Chem. Soc.*, 2014, **136**, 4066–4074.

[9] M. Soederberg, B. Dereka, A. Marrocchi, B. Carlotti and E. Vauthey, *J. Phys. Chem. Lett.*, 2019, **10**, year.

[10] T. Yanai, D. P. Tew and N. C. Handy, *Chem. Phys. Lett.*, 2004, **393**, 51–57.

[11] M. J. Frisch, G. W. Trucks, H. B. Schlegel, G. E. Scuseria, M. A. Robb, J. R. Cheeseman, G. Scalmani, V. Barone, G. A. Petersson, H. Nakatsuji, X. Li, M. Caricato, A. V. Marenich, J. Bloino, B. G. Janesko, R. Gomperts, B. Mennucci, H. P. Hratchian, J. V. Ortiz, A. F. Izmaylov, J. L. Sonnenberg, D. Williams-Young, F. Ding, F. Lipparini, F. Egidi, J. Goings, B. Peng, A. Petrone, T. Henderson, D. Ranasinghe, V. G. Zakrzewski, J. Gao, N. Rega, G. Zheng, W. Liang, M. Hada, M. Ehara, K. Toyota, R. Fukuda, J. Hasegawa, M. Ishida, T. Nakajima, Y. Honda, O. Kitao, H. Nakai, T. Vreven, K. Throssell, J. A. Montgomery Jr., J. E. Peralta, F. Ogliaro, M. J. Bearpark, J. J. Heyd, E. N. Brothers, K. N. Kudin, V. N. Staroverov, T. A. Keith, R. Kobayashi, J. Normand, K. Raghavachari, A. P. Rendell, J. C. Burant, S. S. Iyengar, J. Tomasi, M. Cossi, J. M. Millam, M. Klene, C. Adamo, R. Cammi, J. W. Ochterski, R. L. Martin, K. Morokuma, O. Farkas, J. B. Foresman and D. J. Fox, *Gaussian 16 Rev. B.01*, 2016.

[12] B. Dereka, A. Rosspeintner, M. Krzeszewski, D. T. Gryko and E. Vauthey, *Angew. Chem. Int. Ed.*, 2016, **55**, 15624–15628.
